# Supplementary material for: The nexus between the attitudes and self-concepts of gifted students in an Arab context
Source: PLoS One. 2024 Jun 13;19(6):e0304957. doi: 10.1371/journal.pone.0304957 (PMC11175483; doi:10.1371/journal.pone.0304957)
Supplement: Attachment — Submitted filename: PONE-D-24-06290_R1.pdf [file pone.0304957.s002.pdf]

PLOS ONE

The Nexus Between Attitudes and Self-Concept of Gifted Students in an Arab Context

--Manuscript Draft--

**Manuscript Number:**	PONE-D-24-06290R1
**Article Type:**	Research Article
**Full Title:**	The Nexus Between Attitudes and Self-Concept of Gifted Students in an Arab Context
**Short Title:**	Gifted students in UAE
**Corresponding Author:**	Maxwell Peprah Opoku, PhD, MSc, MSc, BA<br>United Arab Emirates University<br>Al Ain, UNITED ARAB EMIRATES
**Keywords:**	school; gifted and talented students; gifted education; theory of planned behavior; well-being
**Abstract:**	Background: The research literature has reported the need for educators to develop suitable programs geared toward the nurturance of gifted students. Although some non-Western countries have adopted policies to foster the growth of exceptional students, their contributions to research in gifted education are limited. To expand the literature on gifted education, this study was guided by Ajzen's theory of planned behavior to explore the attitudes and self-conceptions of gifted and talented students in the United Arab Emirates.<br>Methods: The revised Self-Perception Profile for Children and Opinions About the Gifted and Their Education scales were used to collect data from 150 high school students in Abu Dhabi, one of seven states (emirates) in the United Arab Emirates. AMOS software version 29 was used to conduct confirmatory factor and path analyses. To evaluate the differences between the background variables on attitudes and self-concepts, t-tests were calculated using SPSS software version 28.<br>Results: The study found a relationship between the attitudes and self-concepts of students regarding their participation in enrichment programs. The hypothesized moderation effect of demographic variables on the relationship between attitudes and self-concepts was not supported.<br>Conclusion: The study's limitations and implications for educators in the United Arab Emirates to prioritize programs geared toward developing the attitudes and self-concepts of gifted students are discussed in detail.
**Order of Authors:**	Maxwell Peprah Opoku, PhD, MSc, MSc, BA
	Ashraf Moustafa
	Negmeldin Alsheikh, PhD
	Noora Anwahi
	Mariam Aljaberi
	Thara Alkateri
	Aysha Almeqbaali
	Hala Elhoweris
**Response to Reviewers:**	Please see attached
**Additional Information:**	
**Question**	**Response**
**Financial Disclosure**<br><br>Enter a financial disclosure statement that describes the sources of funding for the work included in this submission. Review the submission guidelines for detailed	Yes

Powered by Editorial Manager® and ProduXion Manager® from Aries Systems Corporation

requirements. View published research articles from *PLOS ONE* for specific examples.<br><br>This statement is required for submission and **will appear in the published article** if the submission is accepted. Please make sure it is accurate.<br><br>**Funded studies**<br>Enter a statement with the following details:<br>• Initials of the authors who received each award<br>• Grant numbers awarded to each author<br>• The full name of each funder<br>• URL of each funder website<br>• Did the sponsors or funders play any role in the study design, data collection and analysis, decision to publish, or preparation of the manuscript?<br><br>Did you receive funding for this work?	
Please add funding details.<br>as follow-up to "**Financial Disclosure**<br><br>Enter a financial disclosure statement that describes the sources of funding for the work included in this submission. Review the submission guidelines for detailed requirements. View published research articles from *PLOS ONE* for specific examples.<br><br>This statement is required for submission and **will appear in the published article** if the submission is accepted. Please make sure it is accurate.	Office of the Associate Provost for Research at United Arab Emirates University.

Powered by Editorial Manager® and ProduXion Manager® from Aries Systems Corporation

**Funded studies**<br>Enter a statement with the following details:<br>• Initials of the authors who received each award<br>• Grant numbers awarded to each author<br>• The full name of each funder<br>• URL of each funder website<br>• Did the sponsors or funders play any role in the study design, data collection and analysis, decision to publish, or preparation of the manuscript?<br><br>Did you receive funding for this work?"	
Please select the country of your main research funder (please select carefully as in some cases this is used in fee calculation).<br>as follow-up to "**Financial Disclosure**<br><br>Enter a financial disclosure statement that describes the sources of funding for the work included in this submission. Review the submission guidelines for detailed requirements. View published research articles from *PLOS ONE* for specific examples.<br><br>This statement is required for submission and **will appear in the published article** if the submission is accepted. Please make sure it is accurate.<br><br>**Funded studies**<br>Enter a statement with the following details:<br>• Initials of the authors who received each award<br>• Grant numbers awarded to each author<br>• The full name of each funder<br>• URL of each funder website<br>• Did the sponsors or funders play any role in the study design, data collection and analysis, decision to publish, or preparation of the manuscript?<br><br>Did you receive funding for this work?"	UNITED ARAB EMIRATES - AE
**Competing Interests**	The authors declare that they have no competing interests.

Powered by Editorial Manager® and ProduXion Manager® from Aries Systems Corporation

Use the instructions below to enter a competing interest statement for this submission. On behalf of all authors, disclose any competing interests that could be perceived to bias this work—acknowledging all financial support and any other relevant financial or non-financial competing interests.<br><br>This statement is required for submission and **will appear in the published article** if the submission is accepted. Please make sure it is accurate and that any funding sources listed in your Funding Information later in the submission form are also declared in your Financial Disclosure statement.<br><br>View published research articles from *PLOS ONE* for specific examples.<br><br>**NO authors have competing interests**<br><br>Enter: *The authors have declared that no competing interests exist.*<br><br>**Authors with competing interests**<br><br>Enter competing interest details beginning with this statement:<br><br>*I have read the journal's policy and the authors of this manuscript have the following competing interests: [insert competing interests here]*<br><br>* typeset	
**Ethics Statement**<br><br>Enter an ethics statement for this submission. This statement is required if the study involved:<br><br>• Human participants<br>• Human specimens or tissue<br>• Vertebrate animals or cephalopods<br>• Vertebrate embryos or tissues	All methods were performed in accordance with relevant guidelines and regulations. The Institutional Review Committee of the United Arab Emirates University approved this study and its protocols [ERS_2021_8436]. Each study participant was informed of the purpose, method, expected benefit, and risk of the study. They were also informed of their full right to not participate in or withdraw from the study at any time. Written informed consent was obtained from parents of all the study participants.

Powered by Editorial Manager® and ProduXion Manager® from Aries Systems Corporation

• Field research<br><br>Write "N/A" if the submission does not require an ethics statement.<br><br>General guidance is provided below. Consult the submission guidelines for detailed instructions. **Make sure that all information entered here is included in the Methods section of the manuscript.**	

Powered by Editorial Manager® and ProduXion Manager® from Aries Systems Corporation

**Format for specific study types**<br><br>**Human Subject Research (involving human participants and/or tissue)**<br>• Give the name of the institutional review board or ethics committee that approved the study<br>• Include the approval number and/or a statement indicating approval of this research<br>• Indicate the form of consent obtained (written/oral) or the reason that consent was not obtained (e.g. the data were analyzed anonymously)<br><br>**Animal Research (involving vertebrate animals, embryos or tissues)**<br>• Provide the name of the Institutional Animal Care and Use Committee (IACUC) or other relevant ethics board that reviewed the study protocol, and indicate whether they approved this research or granted a formal waiver of ethical approval<br>• Include an approval number if one was obtained<br>• If the study involved *non-human primates*, add *additional details* about animal welfare and steps taken to ameliorate suffering<br>• If anesthesia, euthanasia, or any kind of animal sacrifice is part of the study, include briefly which substances and/or methods were applied<br><br>**Field Research**<br><br>Include the following details if this study involves the collection of plant, animal, or other materials from a natural setting:<br>• Field permit number<br>• Name of the institution or relevant body that granted permission	
**Data Availability**<br><br>Authors are required to make all data underlying the findings described fully available, without restriction, and from the time of publication. PLOS allows rare exceptions to address legal and ethical concerns. See the PLOS Data Policy and FAQ for detailed information.	No - some restrictions will apply

A Data Availability Statement describing where the data can be found is required at submission. Your answers to this question constitute the Data Availability Statement and **will be published in the article**, if accepted.<br><br>**Important:** Stating 'data available on request from the author' is not sufficient. If your data are only available upon request, select 'No' for the first question and explain your exceptional situation in the text box.<br><br>Do the authors confirm that all data underlying the findings described in their manuscript are fully available without restriction?	
**Describe where the data may be found in full sentences. If you are copying our sample text, replace any instances of XXX with the appropriate details.**<br><br>• If the data are **held or will be held in a public repository**, include URLs, accession numbers or DOIs. If this information will only be available after acceptance, indicate this by ticking the box below. For example: *All XXX files are available from the XXX database (accession number(s) XXX, XXX.).*<br>• If the data are all contained **within the manuscript and/or Supporting Information files**, enter the following: *All relevant data are within the manuscript and its Supporting Information files.*<br>• If neither of these applies but you are able to provide **details of access elsewhere**, with or without limitations, please do so. For example:<br><br>*Data cannot be shared publicly because of [XXX]. Data are available from the XXX Institutional Data Access / Ethics Committee (contact via XXX) for researchers who meet the criteria for access to confidential data.*<br><br>*The data underlying the results presented in the study are available from (include the name of the third party*	Data cannot be shared publicly because of ethical reasons. Data are available from the United Arab Emirates University's Ethics Committee (contact via research.office@uaeu.ac.ae) for researchers who meet the criteria for access to confidential data.

and contact information or URL).<br>• This text is appropriate if the data are owned by a third party and authors do not have permission to share the data.<br><br>* typeset	
Additional data availability information:	

Powered by Editorial Manager® and ProduXion Manager® from Aries Systems Corporation

Manuscript

Click here to access/download;Manuscript;submission_final_final_clean.docx

The Nexus Between the Attitudes and Self-Concepts of Gifted Students in an Arabian Context

Maxwell Peprah Opoku[1], Ashraf Moustafa[1], Negmeldin Alsheikh[2], Noora Anwahi[1], Mariam Aljaberi[1], Thara Alkhateri[1], Aysha Almeqbaali[1], Hala Elhoweris[1]

[1]Department of Special and Gifted Education, United Arab Emirates University, Al Ain, United Arab Emirates

[2]Curriculum & Methods of Instruction, United Arab Emirates University, Al Ain, United Arab Emirates

Corresponding author: Dr Maxwell Peprah Opoku (Maxwell.p@uaeu.ac.ae), Assistant Professor, Special Education Department, P. O. Box 15551, Al-Ain–United Arab Emirates.

Abstract

Background: The research literature has reported the need for educators to develop suitable programs geared toward the nurturance of gifted students. Although some non-Western countries have adopted policies to foster the growth of exceptional students, their contributions to research in gifted education are limited. To expand the literature on gifted education, this study was guided by Ajzen's theory of planned behavior to explore the attitudes and self-conceptions of gifted and talented students in the United Arab Emirates.

Methods: The revised Self-Perception Profile for Children and Opinions About the Gifted and Their Education scales were used to collect data from 150 high school students in Abu Dhabi, one of seven states (emirates) in the United Arab Emirates. AMOS software version 29 was used to conduct confirmatory factor and path analyses. To evaluate the differences between the background variables on attitudes and self-concepts, t-tests were calculated using SPSS software version 28.

Results: The study found a relationship between the attitudes and self-concepts of students regarding their participation in enrichment programs. The hypothesized moderation effect of demographic variables on the relationship between attitudes and self-concepts was not supported.

Conclusion: The study's limitations and implications for educators in the United Arab Emirates to prioritize programs geared toward developing the attitudes and self-concepts of gifted students are discussed in detail.

Keywords: school, gifted and talented students, gifted education**,** theory of planned behavior, well-being

Introduction

The need to develop suitable programs for gifted and talented (GT) students has been well explored [1,2]. There is a consensus that GT students can become highly productive citizens who contribute to enriching the communities in which they live by finding solutions to social problems [1,3]. It has been suggested that GT students should be enrolled in appropriate programs that encourage their potential and ability to engage in social problem solving [2,3]. To ensure that GT students derive the maximum benefit from such programs, researchers have paid attention to their self-concepts [4-6] and attitudes toward participating in them [7-10]. Such studies, however, have a relatively limited scope because most have been undertaken in highly developed countries, such as the United States [11,12]. Since GT programs are potentially influenced by the culture and way of life in a given context [13-15], this study aimed to explore the relationship between the attitudes and self-concepts of GT students in a novel Arabian context, the United Arab Emirates (UAE).

The GT concept is not without controversy, and there are two main worldviews regarding giftedness. The first associates giftedness with individuals who excel academically and score at least 140 on IQ testing. This concept of giftedness has been criticized as narrow, restrictive, and favoring a particular group of students from privileged backgrounds [14-16]. Stack et al. [16] argued that defining giftedness according to cognitive ability limits or excludes the enrollment of students from diverse or minority cultural backgrounds in gifted programs. They pointed out that students whose first language is not English are at a disadvantage in enrolling in such programs because the instruments and measurements used to identify GT students are primarily in English. The second worldview is more culture-based and argues that GT is not only related to cognitive ability but also to individuals' ability to manage human relationships [16-18]. Consequently,

there have been calls for the inclusion of informal processes in the concept of GT [13-16]. Even though the GT lens has a limited scope in the UAE and other comparable cultural contexts, there is an ongoing quest for best practices and strategies to promote the development of GT students in these countries [19].

The placement of GT students in programs has been described as labeling [5,11], and there are diverse views concerning ability groupings. For example, one school of thought contends that the identification and subsequent placement of GT students in gifted programs builds their confidence and elicits their best, resulting in improved behavior [11]. The advantages of ability groupings have spurred educators to recognize and place GT students in programs that will boost their development. Conversely, researchers have contended that ability grouping such as enrichment programs is counterproductive as it drives a wedge between those identified as GT and others [5]. As a result, it is challenging for GT students and their peers to form healthy relationships in the same classroom. In addition, some GT students might be complacent because they feel advantaged and unchallenged to work hard to reach their potential [1]. However, despite the risks of identifying and grouping GT students, educators and policymakers should develop initiatives that support them to maximize their potential. GT students will benefit the most from placement in programs that allow their gifts to unfold and fully develop.

Western countries have more experience in developing gifted programs compared with non-Western countries. While the UAE has developed gifted programs for GT students [19], no attempt has been made to understand the attitudes and self-concepts of students enrolled in such programs. The overarching aim of the current study was to investigate the relationship between the attitudes and self-concepts of GT students toward their inclusion in gifted programs.

Theoretical Framework

This study was guided by Ajzen's [20-22] theory of planned behavior (TPB), a major theory used to assess human behavior in societies [23]. The TPB expands on the theory of reasoned action, arguing that behavior is a product or interplay of two related beliefs, behavioral and normative, which impact the intention toward a given behavior [20,24]. Normative beliefs, in contrast to behavioral beliefs, refer to the effect or support of important others on one's intention to perform an action. Behavioral beliefs pertain to one's view of a certain behavior [22]. However, Ajzen [20] later expanded his theory to include a third variable, control beliefs, which might directly impact one's actual behavior. Control beliefs refer to a person's confidence in their capacity to complete a given task or behavior [20,21]. The three related beliefs combine to predict one's intention toward a given behavior. However, although the TPB has been used to guide studies on inclusive education for students with disabilities [22,23], it has rarely been applied to studies of GT students.

For the purpose of this study, the tenets of TPB were adopted. According to Ajzen [20], these tenets develop into variables that predict human behavior. For example, behavioral beliefs develop into attitudes toward a given behavior, normative beliefs develop into subjective norms, and control beliefs develop into perceived behavioral control (used as a proxy for self-concept in this study). In this study, attitudes were operationalized as GT students' perceptions of being categorized and participating in gifted programs. Additionally, subjective norms were explained as the effect or external influence on GT students' involvement in gifted programs. Moreover, self-concept is defined as GT students' understanding of their personalities and confidence in their abilities. According to expectancy-value logic, favorable attitudes toward the three intention components are critical for achieving a desirable outcome [21,22]. Specifically, favorable

attitudes, subjective norms, and positive self-concepts are vital to GT students' success. In other studies on teachers' attitudes toward students with disabilities using the TPB, researchers have reported that favorable attitudes and self-concepts contribute to effective classroom practices [25,26].

Ajzen [20,21] further suggested that the cumulation of subjective norms, attitudes, and self-efficacy explains one's intention to perform a given behavior. The focus of this study, however, was on people's attitudes and self-concepts, because they have direct control over these. Ajzen [20] suggested a relationship between students' attitudes and self-concepts and reasoned that they need to possess favorable attitudes and self-concepts before engaging in a given behavior. In this study, a relationship between attitude and self-concept was proposed. This hypothesis has been tested and supported in studies on inclusive education [25,26].

Ajzen [21] also suggested that the background variables of individuals involved in a given behavior could further explain their intentions. Therefore, human behavior researchers should pay close attention to demographic characteristics and their effects on individual actions or inactions. In this study, it was predicted that background variables could influence the link between attitude and self-concept. In inclusive education research, the TPB has been used to study the association between demographic variables and intentions toward teaching practices [25,26]. However, these studies have yielded contradictory results, and contextual studies of intention are therefore required to develop insights and influence teaching practices. Against this backdrop, in this study, TPB was used as a lens to study the relationship between the attitudes and self-concepts of GT students in the UAE.

Attitudes of Gifted Students

In this study, attitudes refer to students' perceptions of being in a gifted program. According to Gagné [27], understanding attitudes toward gifted education is imperative to develop baseline information about the effectiveness of practices in schools. However, studies on attitudes toward gifted education have been limited mainly to preservice [28] or in-service teachers [12,29]. A large study in Ireland examined teachers' attitudes toward teaching GT students. Although the teachers indicated that they supported gifted education and used specialists to support the teaching of GT students, they had an unfavorable attitude toward grade acceleration. Similarly, in a Swedish study conducted by Ivarsson [30], principals rated their attitudes using the Opinions About the Gifted and Their Education (OGE) scale developed by Gagné and Nadeau [31]. The principals' perceptions of gifted education ranged from negative to ambivalent. However, it is apparent that GT students have not been given many opportunities to share their attitudes toward being in a gifted program.

There is a general consensus that children are "rights bearers" who have the capacity to share or contribute to issues concerning their well-being. Unfortunately, studies on the attitudes of students, such as those in secondary schools, toward their participation in gifted programs are very few. Only a small body of literature has looked at the attitudes of GT students [32] toward gifted programs such as environmental [9,10] and science programs [7,8]. In Turkey, Özarslan [9] conducted studies of GT students participating in a gifted program organized by the Science and Art Center (Bilsem). Both surveys revealed comparable results, demonstrating students' overall positive attitudes toward plants and recycling. However, in the latter study, there was no discernible difference between GT students in terms of age, gender, grade, or years of attendance. In Singapore, studies by Caleon [7] and Lang [8] showed a relationship between

intellectual ability and students' attitudes toward science. Caleon [7] reported that when asked about pursuing science in the future, students with above-average academic abilities and male students were more inclined to respond positively than other groups. Lang [8] discovered that gifted female students had more favorable attitudes toward their interactions with chemistry teachers than male students. Although these findings provide valuable insight into GT students' attitudes toward specific subjects, there are no comparable findings from the UAE.

Previous Studies on GT Students' Self-Concepts

Self-concept has been defined as students' confidence in their abilities and potential. There is a link between student well-being and academic performance [33,34], and underlying this relationship is the self-concept. Researchers have suggested that students with high academic achievement possess positive self-concepts [35]. This finding has highlighted the need for education systems to develop positive self-concepts among students. Consequently, many studies have compared the self-concepts of GT and non-GT students [11,12,30,35-37]. These studies have yielded contradictory findings. Whereas some studies have reported no difference in self-concepts between gifted and non-GT studies [33,36], other studies have shown different results [12, 37]. For instance, in a study conducted across 28 school districts in the United States, McCoach and Rinn [12] indicated a variance between the self-concepts of underachieving and high-achieving students. They reported that GT students scored higher on goal valuation and self-regulation compared to their underachieving counterparts. However, one area of consensus in the research is that gifted and non-gifted students differ in their academic self-concepts [33]. Therefore, to expand the discussion on the importance of self-concept in academic performance, more comparative studies between GT students and non-GT students are needed to inform

educational policy and practice. However, in the UAE, there is no information about the self-concepts of GT students compared with those of their non-gifted counterparts.

Researchers have paid attention to studying the self-concepts of GT students [32]. Many studies have reported differences between students based on their self-concept [4-6,12,33,38,39]. For instance, a review paper by Litster and Roberts [33] compared the differences between GT and non-GT students. On athletics and perceived looks, GT students reportedly scored lower than non-GT students. Gifted students, however, outperformed non-gifted students in both academic performance and perceived behavior. Since the UAE is currently developing its system to encourage gifted education [19], more scholarly attention needs to be paid specifically to students involved in gifted programs beyond comparison studies with students who are not involved in gifted programs.

Overview of the Study

Following the 2008 educational policy in the UAE, the Ministry of Education formulated the country's educational strategy goal of 2010–2020, which was a student-centered approach to education [19,40]. The strategic plan emphasized that every student with an exceptional learning need requires individualized education to enable them to achieve their learning goals. Additionally, the Ministry of Education launched the flagship gifted education enrichment program entitled the "Development of Gifted and Talented Students' Skills." During the 2014 calendar year, the program underwent restructuring and was rebranded the "Integrated System to Identify and Care for Talents." Schools were expected to encourage high achievers to investigate social problems and use innovative problem-solving approaches to solve them [40].

These policy initiatives have contributed to the formation of interest groups and associations supporting the education of GT students across the UAE. Notable is the Hamdan Foundation, which promotes gifted education, teacher development, public awareness, parental training, and the implementation of gifted programs in the UAE [19,40]. Although empirical evidence has revealed discrepancies and inadequate enactment of gifted education in the UAE [19], there is a dearth of empirical studies investigating students' learning experiences in these programs.

Current Study

Based on Ajzen's [20] conception of the TPB, a proposition was made in this study with respect to the relationship between self-concepts and attitudes. According to Gagné [27], the extent to which aspects of attitude predict other variables, such as self-concept, has not received much scholarly attention. In view of this, we hypothesized the following:

Hypothesis I: GT students' self-concepts will predict their attitudes toward their involvement in gifted programs (see Figure 1).

Additionally, demographic variables have been identified as vital in efforts to understand the intentions toward a given behavior. For example, in the UAE, gender is a major factor in the grouping of secondary schools according to their high-ability areas, and, typically, secondary school students attend single-sex classrooms. Consequently, their enrollment in gifted programs has followed a gendered pattern. In this study, gender and high-ability area were selected as variables of interest to explore their impact on the relationship between students' self-concepts and their attitudes toward gifted programs:

Hypothesis II: Demographic variables will be a significant moderator of the attitudes and self-concepts of gifted students.

The available evidence has indicated that GT students' attitudes and self-concepts have been studied individually. However, there is little or no empirical research on the relationship between GT students' attitudes and self-concepts. Studies examining students' attitudes and self-concepts are also sporadic in Arabian contexts. Therefore, there is a need to examine the relationship between attitudes and self-perceptions among GT students in the UAE. This study aimed to answer the following research questions:

1. What are the differences between GT students in demographic variables, attitudes, and self-concepts?
2. Do GT students' self-concepts predict their attitudes toward participating in gifted programs in the UAE?
3. Do demographic variables moderate the self-concepts and attitudes of GT students toward participating in gifted programs in the UAE?

Methods

Design

The study entailed cross-sectional, descriptive research that aimed to capture the self-concepts of students at a given time [41]. The design was appropriate as it enabled the study of students' attitudes and self-concepts at a given point in time.

The participants in this study were GT high school students nominated by their schools to participate in an enrichment program. In the UAE, there is a three-tier educational structure:

cycle one (pre-school to grade 4), cycle two (grades 5–8), and cycle three (grades 9–12). While public schools are managed by the Emirates Schools Establishment, they are also supervised by the Abu Dhabi Department of Education and Knowledge (ADEK). In this study, GT students were identified based on their performance on IQ tests conducted in the schools. GT students participate in projects related to the science, technology, engineering and mathematics (STEM) areas of high ability. For instance, some of the students have been involved in the Maker's Real Engagement in Active Problem-Solving project [42] on environmental pollution.

Table 1. Difference between background variables, attitudes, and self-concept

	Sample	Attitude	Self-concept
**Gender**			
Male	37 (25%)	3.62 (.54)	3.77 (.65)
Female	113 (75%)	3.66 (.55)	3.66 (.61)
t		-.34	.91
Cohen's d		.06	.17
**Age**			
10–15 years	38 (25%)	3.62 (.56)	3.66 (.57)
16–18 years	112 (75%)	3.66 (.54)	3.69 (.64)
t		-.33	-.27
Cohen's d		.06	.05
**Grade Level**			
Intermediary level (Grades 7–9)	40 (27%)	3.65 (.65)	3.75 (.66)
High School (Grades 10–12)	108 (73%)	3.65 (.51)	3.66 (.61)
t		.009	.08
Cohen's d		.002	.15
**Area**			
STEM areas	110 (73%)	3.64 (.55)	3.69 (.66)
Other areas	40 (27%)	3.68 (.54)	3.67 (.51)
t		-.37	.15
Cohen's d		.07	.03

STEM: science, technology, engineering and mathematics

Simple random sampling was used to recruit the study participants from the sampling frame [43]. Each student involved in the enrichment program was invited to participate in this

study. The current study drew GT students from private schools, as they were the main schools running enrichment programs for GT students. The decision to include students from private schools was based on ADEK granting its permission to include students on their high-ability roster. Prior consent was sought from all parents whose children were on the ADEK register. Invitations were sent via email to all GT students participating in the enrichment program. A total of 150 students took part (see Table 1).

Data Collection

This study forms part of a larger project that sought to understand the well-being of GT students participating in an enrichment program in the UAE [44]. The study was approved by the Social Sciences Ethics Review Committee of the United Arab Emirates University (ERS_2021_8436).

The survey was made up of three sections. The first section collected demographic data (gender, age, grade level, and high-ability area(s)), which were included based on a review of the previous literature [8-10,12].

The ensuing parts of the instrument were published scales related to the components (attitudes and self-concepts used as a proxy for self-efficacy) of Ajzen's [20] TPB, which was adapted for this study. Specifically, the second part [45] used a revised Self-Perception Profile for Children (SPPC) scale, which has been used extensively to study the self-concept.

The scale is made up of 34 items scored on a 5-point scale: strongly disagree (1) to strongly agree (5). The scale comprises six subscales (scholastic competence [students' rating of their academic ability], n = 5; social acceptance [ability of students to develop healthy social relationships], n = 6; athletic competence [competence of students in extracurricular sporting

activities], $n = 6$; physical appearance [extent to which students accept themselves], $n = 5$; behavioral conduct [students' ratings of their emotional regulations], $n = 6$; and global self-worth [defined as how students appreciate and embrace themselves], $n = 6$).

The third part was a revised 20-item OGE scale [31]. The OGE scale was adopted for this study because it has been used extensively to study teachers' attitudes [27,32]. However, in Gagné's [27] view, the instrument needed to be more efficient and concise to capture the core components of attitudes. Based on feedback from expert reviewers, the authors settled on the revised instrument, which is short and captures the important aspects of attitude that can be reported by secondary school students. Additionally, this appears to be the first time the OGE has been implemented for students in a non-Western context; thus, we needed to use a version that students could easily comprehend. These justifications informed the use of the abridged version of the original instrument. Responses to the measure, which consists of three subscales (support, acceleration, and elitism), are anchored on a 5-point Likert scale ranging from strongly disagree (1) to strongly agree (5).

The instrument consisted of three subscales. First, support for gifted education is comprised of 10 items and refers to the extent to which participants agree with their involvement in a special program for the gifted. Elitism, which comprises six items, assesses participants' beliefs as to whether being involved in a gifted program represents favorable or preferential treatment given to gifted students. Acceleration, constituting four items, measures beliefs about the acceleration of gifted students in schools [12].

Three specialists were given the questionnaire to assess and remark on its suitability for data gathering. This process is called the Delphi approach [46], which is a review of instruments by experts with in-depth knowledge who evaluate the suitability of a questionnaire before its

implementation. Two versions of the instrument were given to the experts so that they could determine the version most suitable for data collection. There was consensus among the specialists on the use of the short version for data collection for this study. Following this, the instrument was piloted with eight GT students who were not considered for participation to determine whether it was clear and simple to understand.

An official invitation was sent to ADEK seeking permission to conduct this study. The parents of children on the high-ability roster gave ADEK their consent before their email contacts were released to the research team. The parents were informed of the study's goals and provided a breakdown of its methodology. They were also assured that no identifiable information about their children would be used or shared with anyone outside the research team. The parents were further informed that their children would not be incentivized to participate in this study. The children whose parents responded positively provided informed consent for participation in this study.

Google Forms was employed for data collection due to the mobility challenges imposed by the COVID-19 pandemic. There were Arabic and English translations of the items on the instrument. The data were collected from May 2022 to July 2022. The duration of the survey completion was 15–25 minutes.

Data Analysis

The data were cleaned in Microsoft Excel before being imported to the Statistics Package for Social Science (SPSS) version 28 for analysis. The initial computation of missing data using missing at-random tests showed a range of 0.6% to 1.7%. Following this, the missing data were imputed. After that, a normality test was performed to determine whether the data were

appropriate for parametric analysis. The results of the Kolmogorov–Smirnov and Shapiro–Wilk tests were insignificant, suggesting the normality of the data for parametric testing [47].

While the SPPC was validated in an earlier study [44], the OGE was used for the first time in the current context and thus, tt was necessary to assess its construct validity. Consequently, confirmatory factor analysis (CFA) was computed to understand OGE's dimensionality. The following goodness-of-fit indices were used: a chi-square value of less than 0.5, a comparative fit index (CFI) and a Tucker-Lewis index (TLI) of 0.90 or greater, a root square error of approximation (RMSEA), a standard root mean square residual (SRMR) of at least 0.08, and a regression weight of at least 0.50 [48,49]. However, if the model did not meet the above thresholds, modification indices were observed to eliminate items with high and erroneous covariances [50].

The computation CFA for the 20-item OGE scale showed a poorly fit model: chi-square = 3.02, CFI = 0.60, TLI = 0.53, RMSEA = .012, and SRMR = 0.11. The items on the elitism subscale had poor loadings (items 11, 12, 13, 14, 15, and 16), and thus were deleted from the model. Additionally, half of the items of the support subscale (5, 7, 8, 9, and 10) had factor loadings below the cutoff and were removed from the model (see Figure 1). The rerun reached acceptable indices: chi-square = 1.12, CFI = 0.99, TLI = 0.98, RMSEA = 0.03, and SRMR = 0.05. However, there was no correlation between the subscales support and acceleration ($r = -.08$).

The instrument's reliability was assessed using Cronbach's alpha: attitude (0.60) and self-concept (0.90). The outcomes confirmed that the tool was suitable for reporting the study.

Figure 1. Confirmatory factor analysis for an opinion about the gifted and their education

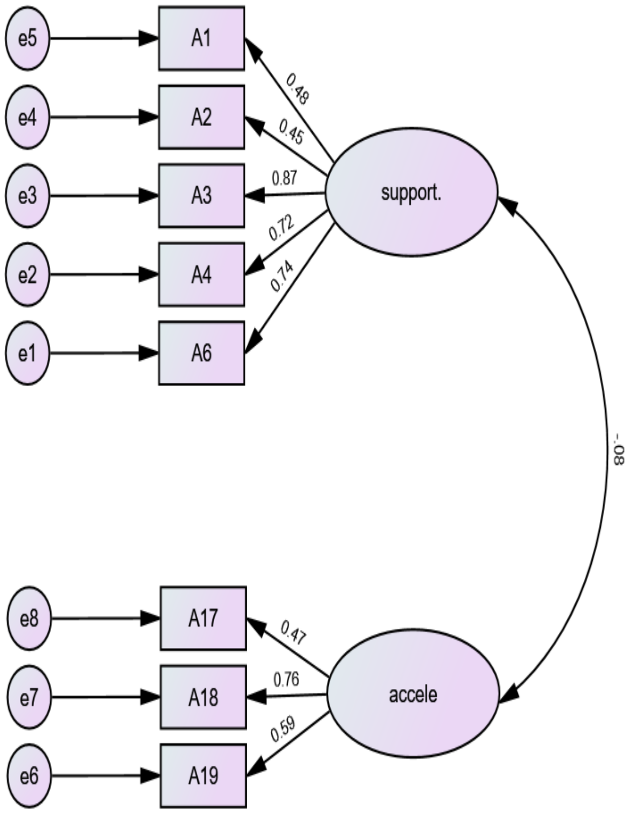

Note: accele = acceleration; support = support for gifted programs

For Research Question 1, which concerned the effects of GT students' demographics on attitudes and self-concepts, t-tests were used because all the demographics had two levels. An assumption of variance homogeneity was observed to ensure that it was not violated [47].

To answer Research Question 2, direct path analysis, a form of structural equation modeling, was used to test the relationship between attitudes and self-concepts. Path analysis

was used because it is appropriate for testing hypotheses [50], such as the assumed relationship between attitude and self-concept.

A preliminary CFA was used to determine the relationship between overall attitudes and self-perceptions, and the results were interpreted using the following thresholds: small (0.10–0.30), moderate (0.31–0.50), and large (≥ 0.51) [47]. The contribution of self-concept to the variance in attitudes was computed using path analysis. Based on Gagné's [27] suggestion that researchers investigate the types of attitudes and the specific behaviors they influence, path analysis was conducted at the subscale levels. To ensure that the model was appropriate, the fit indices stated above were observed.

For Research Question 3, the two demographic variables of interest—gender and students' ability areas—were added to the earlier path analysis model to assess their interaction effects on the association between attitudes and self-concepts. Both bootstrap and bias-corrected confidence intervals were set at 500 and 95%, respectively. In cases where there was a significant moderation effect, Andrew Hayes's [51] PROCESS model was used to explore the direct effect of the moderators on attitudes and self-concepts.

Results

Association Between Demographics, Attitude, and Self-Concept

The computation of mean scores showed students' neutrality on attitudes (M = 3.65; SD = 0.54) and self-concepts (M = 3.69; SD = 0.62). Additionally, the mean scores for self-concept showed that participants were high on global self-worth (M = 4.12; SD = 0.91) but ambivalent on the remaining subscales (scholastic competence, M = 3.94, SD = 0.74; social acceptance, M =

3.49, *SD* = 1.20; athletic competence, *M* = 3.01, *SD* = 1.11; physical appearance, *M* = 3.42, *SD* = 1.29; behavioral conduct, *M* = 3.93, *SD* = 1.29; see Table 2).

Table 2. Summary of mean scores on self-concept

	**Items**	M	SD
	**Scholastic competence**	3.94	.91
W1	I am good at schoolwork	4.34	.82
W3	I remember lesson easily	3.77	.94
W4	I perform very well in science and mathematics	3.92	.94
W5	I figure out answer easily	3.72	.93
	**Social acceptance**	3.49	1.20
W7	I have a lot of friends	3.71	1.24
W9	I do things with a lot of friends	3.64	1.15
W11	I am very popular in school	3.12	1.21
	**Athletic competence**	3.01	1.11
W12	I am very good at sports	3.32	1.10
W15	I am better than others in sports	2.79	1.15
W17	I am good at both outdoor and indoor sports	2.92	1.07
	**Physical appearance**	3.42	1.29
W18	I am very happy with my physical appearance	3.48	1.22
W19	I am happy with my height and weight	3.31	1.34
W20	I like my body as it is	3.46	1.27
W21	I like my physical appearance as it is	3.42	1.32
	**Behavior conduct**	3.93	1.29
W23	I like my behaviour	4.13	.94
W24	I usually do the right thing	3.77	1.00
W25	I usually do things in the right way	3.83	.89
W28	I behave well all things	3.98	.97
	**Global self-worth**	4.12	.97
W29	I am happy with myself	4.12	.96
W31	I am happy with myself as a person	4.12	.95
W32	I like myself	4.17	.97
W33	I am happy the way I am	4.05	1.01

Concerning the subscale on attitudes, the participants were ambivalent about support (*M* = 3.71, *SD* = 1.02) and acceleration (*M* = 3.56, *SD* = 1.10; see Table 3).

Table 3. Summary of mean scores on attitude towards gifted education

	**Item**	**M**	**SD**
	**Support**	3.71	1.02
A1	I have happy being in a gifted program in my school.	4.11	.91
A2	Most of my family and friends consider me gifted.	2.89	1.33
A3	I consider myself to be gifted	3.91	.94
A4	Most of my family and friends are gifted.	3.97	.94
A6	Our schools offer appropriate special education services for the gifted.	3.65	1.00
	**Acceleration**	3.56	1.10
A17	Most gifted children who skip a grade have difficulties in their social adjustment to a group of older students.	3.69	1.21
A18	Children who skip a grade are usually pressured to do so by their parents.	3.58	.97
A19	When skipping a grade, gifted students miss important ideas	3.93	1.13

To determine whether there was an association between background variables, attitudes, and self-concepts, independent t-tests were conducted. The outcomes revealed no differences in the participants' attitudes or self-perceptions (see Table 1).

Predictors of Attitudes

Path analysis was computed to ascertain whether self-concepts could contribute to the variance in attitudes (see Figure 2). An initial CFA showed a significant correlation between overall attitudes and self-concepts (r = 0.81).

Figure 2. Contribution of self-conception in the variance in attitude

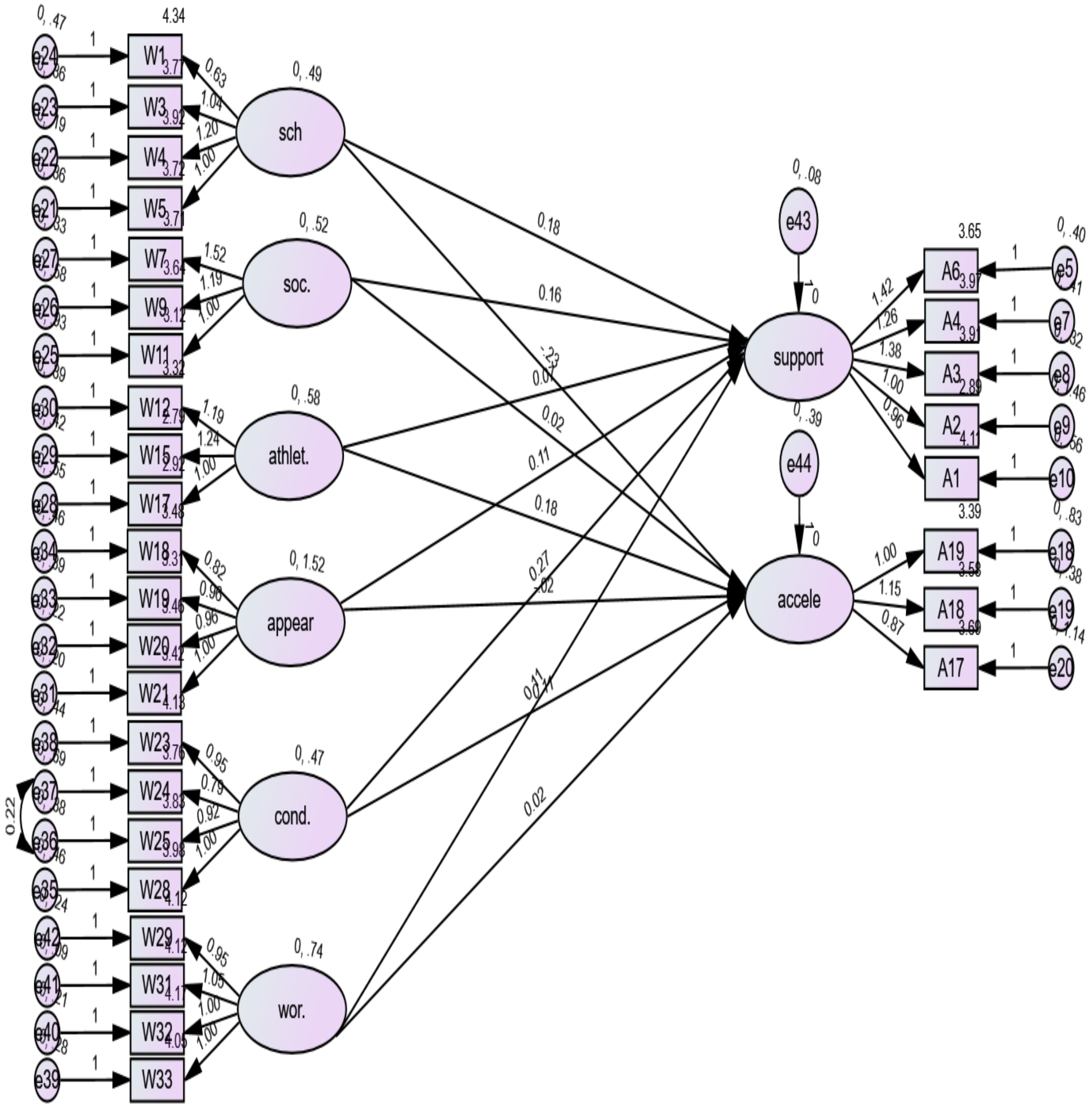

Note: sch = scholastic competence; soc. = social acceptance; athlete. = athletic competence; appear = physical appearance; cond. = behavior conduct; and wor. = global self-worth).

The contribution of self-concept to the variance in attitudes was assessed. First, regarding the attitude subscale of support, the following subscales of self-concept significantly contributed to its variance: scholastic competence ($b = 0.30$, $p = 0.01$), social competence ($b = 0.28$, $p = 0.02$), physical appearance ($b = 0.33$, $p = .005$), behavioral conduct ($b = 0.45$, $p = .004$), and global self-worth ($b = 0.23$, $p = .02$). Second, concerning the attitude subscale of acceleration, only one predictor made a significant contribution to the variance: scholastic competence ($b = -0.24$, $p = .03$).

Moderation

To comprehend the impact of gender and ability area on the relationship between attitude and well-being, moderation analyses were performed. First, gender was operationalized as a moderator to understand its influence on the relationship between acceleration and self-concept. The initial model looked at how gender affected one subscale of attitude and self-concept. Gender had a direct effect only on physical appearance ($b = .03$, $p =. 04$). However, gender did not influence acceleration ($b = -.24$, $p = .52$). Concerning the standardized indirect effect, the results showed that gender had no interaction effect on the relationship between acceleration and self-concept.

Second, the moderation effect of gender on the relationship between the second subscale of attitude, support, and well-being was assessed. Regarding the standardized direct effect, gender had no direct influence on support ($b = -.10$, $p = .55$). Furthermore, the association between support for gifted education and well-being was unaffected by gender.

The interaction effect of a high-ability area on the link between attitude and well-being was also assessed. For instance, the area of high ability had a standardized direct effect on social

competence (b = .08, p = .01) and scholastic competence (b = −.60, p = .01). Additionally, the area of high ability had no direct influence on acceleration (b = −.13, p = .61) and support for gifted education (b = −.22, p = .63). Areas of high ability did not have a standardized indirect effect on the association between attitude and self-concept. Similar findings were observed regarding the degree to which grade level and age moderated the link between attitudes and self-perceptions.

Discussion

In this study, Ajzen's [20] TPB was adopted to understand the relationship between the attitudes and self-concepts of GT students in a novel cultural context, the UAE. The CFA used in the study, whose factorial validity was supported, was used to test the structural validity of the OGE and SPPC scales. The results showed a six-factor structure for the SPPC and a two-factor structure for the OGE. Although modifications were deployed to eliminate reduced items whose erroneous covariances affected the model fit indices [50], the tools were validated in a context other than the usual Western context, where such studies are usually conducted. The validation process involved the observation of numerous parameters (chi-square, CFI, TLI, modifications, etc.) [5,48,50] before ascertaining their validity. The results demonstrate the potential of the SPPC and OGE measures to examine talented students' attitudes and self-perceptions in the UAE and other Arabian countries.

The results of the study supported Hypothesis I and demonstrated a relationship between self-concept and attitude. This finding supports Ajzen's [20] proposition of a relationship between individuals' attitudes and self-concepts in explaining their intention toward a given

behavior. The results are consistent with earlier studies on inclusive education, which revealed a favorable association between people's attitudes and perceptions of their own actions [25,26]. The findings suggest that in the UAE context, as the attitudes of GT students toward participation in gifted programs improve, the students' self-concepts may also improve. The UAE government's top priority is to educate and prepare future generations to support the nation's development [40]. However, achieving this goal relies heavily on GT students, who must be identified and placed in requisite programs where their skills will be nurtured. Unquestionably, if educators make conscious efforts to improve students' attitudes, which might affect GT students' self-perceptions, and vice versa, such a goal could be accomplished.

Although Ajzen [20] argued about the potential for demographic variables to impact intention toward a given behavior, this position was not supported in this study. Background factors were originally thought to have an impact on how GT students in the UAE viewed themselves with respect to their attitudes. However, the findings showed that no demographic variables had an association with or moderated the relationship between attitude and self-concept. This conclusion conflicts with earlier GT studies that found that attitudes and self-perceptions were influenced by demographic factors [6-8]. The study finding is also inconsistent with previous inclusive education research, which reported the influence of demographic variables on attitudes and self-concepts toward learning [25,26]. In the current study, the participants were from private schools only, which could influence the trend identified in this study. Therefore, future studies could examine the attitudes and self-perceptions of GT students in both public and private schools.

One curious finding was the lack of a relationship between the two attitude subscales, support and acceleration. Attitudes explain the disposition of participants toward being identified

and included in gifted programs. Ajzen [20] contended that belief accumulation explains attitudes toward a given behavior, such as being involved in gifted programs. However, in this study, the findings showed no relationship between acceleration and support for gifted programs. Notably, the study's participants engaged in enrichment activities that could have influenced their results. There is a possibility that they were aware of the enrichment program and not the acceleration program. Anecdotal data indicate that many schools in the UAE discourage acceleration, particularly in private institutions. However, the findings indicated that the participants were unsure of support for gifted programs. The UAE government is currently looking at programs to enhance GT students' development [19,40]. This could trigger or expedite discussions among UAE educators on the best methods and support programs for GT students in the country.

The results appear to support the multidimensionality of self-concept in the context of the current investigation. According to Winne et al. [34], self-concept should be studied with a multiple lens to develop a holistic understanding. For example, the effectiveness of studies may be impacted by both classroom procedures and extracurricular activities. The association between the self-concept subscales seems to provide useful guidance for education practices in the UAE, even if the purpose here is not to establish causation. It is useful for educators in the UAE to observe a multiplicity of factors that could negatively impact the confidence of GT students. Indeed, a significant correlation was observed between self-worth and behavior management. The tendency of GT students to exhibit unfavorable behavior has been mentioned in some literature, which might affect how well they succeed [1,2]. In the UAE context, to avert this situation, policymakers might consider developing the self-worth of individuals by building their self-confidence and beliefs, which could positively affect their behavioral conduct.

Conclusion

The study was guided by Ajzen's [20] TPB. The main objective was to investigate the connection between GT students' attitudes and self-perceptions in the UAE context. The TPB [20] makes two important propositions: (a) there is a relationship between the predictors and intentions toward a given behavior, and (b) background variables have an influence on the predictors. In the research on GT students in the UAE, these two hypotheses were put to the test. One proposition was supported by the study's findings, whereas the other was not. The study's findings showed a substantial relationship between GT students' self-concepts and attitudes. Conversely, demographic variables did not influence the relationship between attitudes and self-concepts. In conclusion, the study supports the idea that attitudes and self-concepts are crucial in creating a successful program for GT students in the UAE and other similar settings.

Limitations and Recommendations for Future Research

It is impossible to generalize our findings because the study was constrained in certain ways. First, the researchers recruited GT students from private schools participating in an enrichment program in one of the seven states in the UAE. Hence, the interpretation of the study's findings is limited to students from private schools participating in an enrichment program. However, the UAE is a diverse society with students from varied backgrounds and cultures enrolled in private schools. Moreover, one regulating agency, the Ministry of Education, oversees and approves teaching standards and maintains schools. Although the findings could reflect the situation more generally in the country, future research could extend this study to public schools to compare the attitudes and self-concepts of GT students enrolled in both private

and public schools. Second, the data were collected online, and the opportunity to ask the students questions for clarification may have been limited. Future research could use qualitative techniques to develop thorough insights into the experiences of GT students. Third, the findings are an account of GT students only, and it is uncertain whether non-GT students could share the same level of attitudes and self-concepts. Future research could examine how GT and non-GT students view themselves to develop holistic insights into their self-concepts and attitudes toward gifted education.

Nevertheless, the findings could have implications for policymaking in the UAE and other similar cultural contexts. First, policymakers could consider developing awareness and training programs for GT students geared toward enabling them to understand their contributions to national development. Additionally, GT students could be made more aware of why it is important for them to participate in gifted programs. Furthermore, GT students could be involved in extracurricular activities to help improve their self-concepts. For instance, aside from academic programs, educators could consider organizing athletic programs and related activities for GT students to improve their self-concepts. Third, educators could consider clarifying the scope of gifted programs in the UAE. This could include the criteria for acceleration and fine-tuning acceleration programs, which are conterminous with contextual realities. The UAE is working to enhance students’ well-being, and measures such as those suggested above could assist in fostering an environment that is favorable to the development of GT students.

List of abbreviations

CFA	Confirmatory factor analysis
CFI	Comparative fit index
EFA	Exploratory factor analysis
GT	Gifted and talented
SRMR	Standard root mean square residual
UAE	United Arab Emirates

Declarations

Availability of data statement

The datasets generated and analysed during the current study are not publicly available due to ethical restrictions but are available from the corresponding author upon reasonable request.

Financial disclosure

The study was funded by the Office of the Associate Provost for Research at United Arab Emirates University. The funders had no role in study design, data collection and analysis, decision to publish, or preparation of the manuscript.

Conflict of interest statement

The authors declare that they have no competing interests.

Acknowledgements

We would like to thank the Abu Dhabi Department Knowledge and Education for their support during recruitment and data processing. Our heartfelt gratitude goes to all students and parents who participated in this study.

Authors' contributions

Conceptualisation: Maxwell Peprah Opoku, Ashraf Moustafa, Negmeldin Alsheikh, Noora Anwahi, Mariam Aljaberi, Thara Alkhateri, Aysha Almeqbaali, Hala Elhoweris
Data curation: Maxwell Peprah Opoku, Ashraf Mustafa
Formal analysis: Maxwell Peprah Opoku
Investigation: Maxwell Peprah Opoku,
Methodology: Maxwell Peprah Opoku, Ashraf Moustafa, Negmeldin Alsheikh, Noora Anwahi, Mariam Aljaberi, Thara Alkhateri, Aysha Almeqbaali, Hala Elhoweris
Project administration: Maxwell Peprah Opoku
Resources: Maxwell Peprah Opoku
Writing – original draft: Maxwell Peprah Opoku, Ashraf Moustafa, Negmeldin Alsheikh, Noora Anwahi, Mariam Aljaberi, Thara Alkhateri, Aysha Almeqbaali, Hala Elhoweris
Writing and reviewing the draft: Maxwell Peprah Opoku, Ashraf Moustafa, Negmeldin Alsheikh, Noora Anwahi, Mariam Aljaberi, Thara Alkhateri, Aysha Almeqbaali, Hala Elhoweris

References

1. Heward WL, Ford DY. Gifted and Talented. In William L. Heward Exceptional children: An introduction to Special Education (10th Edition). Pearson; 2013.

2. Merrotsy P. Supporting outstanding learners. In Adrian Ashman (editor), Education for Inclusion and diversity (p. 233-264). Pearson; 2015.
3. Kuo CC, Maker J, Su FL, Hu, C. Identifying young gifted children and Cultivating problem solving abilities and multiple intelligences. Learning and Individual Differences. 2010; 20(4): 365–379.
4. Cross TL, Swiatek MA. Social coping among academically gifted adolescents in a residential setting: A longitudinal study. Gifted Child Quarterly. 2009; 53(1): 25-33. 10.1177/0016986208326554
5. Hoge RD, Renzulli JS. Exploring the link between giftedness and self-concept. Review of Educational Research. 1993; 63(4): 449-465. https://doi.org/10.3102/00346543063004449
6. Košir K, Horvat M, Aram U, Jurinec N. Is being gifted always an advantage? Peer relations and self-concept of gifted students. High Ability Studies. 2016; 27(2), 129–148. https://doi.org/10.1080/13598139.2015.1108186
7. Caleon IS, Subramaniam R. Attitudes towards science of intellectually gifted and mainstream upper primary students in Singapore. Journal of Research in Science Teaching: The Official Journal of the National Association for Research in Science Teaching. 2008; 45(8): 940-954. https://doi.org/10.1002/tea.20250
8. Lang QC, Wong AFL, Fraser Barry J. Teacher-Student Interaction and Gifted Students' Attitudes toward Chemistry in Laboratory Classrooms in Singapore. The Journal of Classroom Interaction. 2005; 40(1): 18-28

9. Özarslan M. Perceptions and attitudes of gifted and talented secondary school students towards plants. Gifted Education International. 2020; 36(3); 266-284. https://doi.org/10.1177/0261429420946735

10. Ugulu I. A quantitative investigation on recycling attitudes of gifted/talented students. Biotechnology & biotechnological equipment. 2015; 29(sup1): S20-S26. https://doi.org/10.1080/13102818.2015.1047168

11. Berlin JE. It's all a matter of perspective: Student perceptions on the impact of being labeled gifted and talented. Roeper Review. 2009; 31(4): 217-223. https://doi.org/10.1080/02783190903177580

12. McCoach DB, Rinn D. Factors that differentiate underachieving gifted students from high-achieving gifted students. Gifted Child Quarterly. 2007; 47(2): 144-154. https://doi.org/10.1177/001698620304700205

13. Callahan CM. Identifying gifted students from underrepresented populations. Theory into Practice. 2005; 44(2): 98-104, https://doi.org/10.1207/s15430421tip4402_4

14. Ford DY. Segregation and the Underrepresentation of Blacks and Hispanics in Gifted Education: Social Inequality and Deficit Paradigms. Roeper Review. 2014; 36(3): pp. 143–154, DOI: 10.1080/02783193.2014.919563

15. Milner HR, Ford DY. Cultural considerations in the underrepresentation of culturally diverse elementary students in gifted education. Roeper Review. 2007; 29(3): 166-173. https://doi.org/10.1080/02783190709554405

16. Stack N, Sutherland M, Smith TA, Tungaraza FD. Creating a space for diverse learners in multifarious contexts. In Taber KS, Sumida, M. International perspectives on Science

education for the gifted: key issues and challenges (pp. 140–153). Taylor and Francis; 2016.

17. Maree JG. Gifted education in Africa. In S. I. Pfeiffer, E. Shaunessy-Dedrick, & M. Foley-Nicpon (Eds.), APA handbook of giftedness and talent (pp. 131–142). American Psychological Association; 2018. https://doi.org/10.1037/0000038-009
18. Ngara C. Gifted education in Zimbabwe. Cogent Education. 2017; 4(1): 1–14. https://doi.org/10.1080/2331186X.2017.1332840
19. AlGhawi MA. Gifted education in the United Arab Emirates. Cogent Education. 2017; 4(1): 1368891. https://doi.org/10.1080/2331186X.2022.2034247
20. Ajzen I. The theory of planned behavior. Organizational Behavior and Human Decision Processes. 1991; 50(2): 179–211. https://doi.org/10.1016/0749-5978(91)90020-T
21. Ajzen I. The theory of planned behaviour: reactions and reflections. Psychology & Health. 2011; 26(9): 1113–1127. https://doi.org/10.1080/08870446.2011.613995
22. Ajzen I. The theory of planned behavior: Frequently asked questions. Human behavior and emerging technologies. 2020; 2(4): 314-324. https://doi.org/10.1002/hbe2.195
23. Cheng EW. Choosing between the theory of planned behavior (TPB) and the technology acceptance model (TAM). Educational Technology Research and Development. 2019; 67: 21-37. https://doi.org/10.1007/s11423-018-9598-6
24. La Barbera F, Ajzen I. Control interactions in the theory of planned behavior: Rethinking the role of subjective norm. Europe's Journal of Psychology. 2020; 16(3); 401. https://doi.org/10.5964/ejop.v16i3.2056
25. Opoku MP, Cuskelly M, Pedersen SJ, Rayner SC. Attitudes and Self-Efficacy as Significant Predictor of Intention of Secondary School Teachers towards the

Implementation of Inclusive Education in Ghana. European Journal of Psychology of Education. 2021; 36: 673-691. https://doi.org/10.1007/s10212-020-00490-5

26. Opoku MP, Cuskelly M, Pedersen SJ, Rayner SC. Applications of the Theory of Planned Behaviour in assessing intentions of teachers towards practicing inclusive education: a scoping review. European Journal of Special Needs Education. 2021; 36(4): 577-592. https://doi.org/10.1080/08856257.2020.1779979
27. Gagné F. Attitudes toward gifted education: Retrospective and prospective update. Psychological Test and Assessment Modeling. 2018; 60(4): 403. https://doi.org/10.1080/02783193.2007.11869221
28. Camcı-Erdogan S. Investigating pre-service gifted education teachers' self-efficacy toward science teaching and scientific attitudes. Eurasian Journal of Educational Research. 2015; 59: 133-148. http://dx.doi.org/10.14689/ejer.2015.59.8
29. Cross TL, Cross JR, O'Reilly C. Attitudes about gifted education among Irish educators. High Ability Studies. 2018; 29(2): 169-189. https://doi.org/10.1080/13598139.2018.1518775
30. Ivarsson L. Principals' perceptions of gifted students and their education. Social Sciences & Humanities Open. 2023; 7(1): 100400. https://doi.org/10.1016/j.ssaho.2023.100400
31. Gagné F, Nadeau L. Opinions about the gifted and their education. Unpublished instrument; 1991.
32. Cross JR, Cross T, Frazier AD. Student and teacher attitudes toward giftedness in a two-laboratory school environment: A case for conducting a needs assessment. NALS Journal. 2013; 5(1): 1-21.

33. Litster K, Roberts J. The self-concepts and perceived competencies of gifted and non-gifted students: A meta-analysis. Journal of Research in Special Educational Needs. 2011; 11: 130–140. doi:10.1111/j.1471-3802.2010.01166.x

34. Winne PH, Woodlands MJ, Wong BY. Comparability of self-concept among learning disabled, normal, and gifted students. Journal of Learning Disabilities. 1982; 15(8): 470–475. https://doi.org/10.1177/002221948201500805

35. Yan K, Haihui Z. A Decade Comparison: Self-Concept of Gifted and Non-Gifted Adolescents. International Education Journal. 2005; 6(2): 224-231.

36. Peperkorn C, Wegner C. Academic self-concept of gifted and nongifted biology students: indications for domain-specific gifted education. Progress in Science Education (PriSE). 2023; 6(2): 6-19. https://doi.org/10.25321/prise.2023.1326

37. Preckel F, Schmidt I, Stumpf E, Motschenbacher M, Vogl K, Scherrer V, Schneider W. High- ability grouping: Benefits for gifted students' achievement development without costs in academic self- concept. Child development. 2019; 90(4): 1185-1201. https://doi.org/10.1111/cdev.12996

38. Rinn AN, Mendaglio S, Moritz Rudasill K, McQueen KS. Examining the relationship between the overexcitabilities and self-concepts of gifted adolescents via multivariate cluster analysis. Gifted Child Quarterly. 2010; 54(1): 3-17. https://doi.org/10.1177/0016986209352682

39. Rudasill KM, Callahan CM. Psychometric characteristics of the Harter Self-Perception Profiles for adolescents and children for use with gifted populations. Gifted Child Quarterly. 2008; 52(1): 70-86. https://doi.org/10.1177/001698620731105

40. Ismail SA, Alghawi MA, AlSuwaidi KA. Gifted education in United Arab Emirates: Analyses from a learning-resource perspective. Cogent Education. 2022; 9(1): 2034247. https://doi.org/10.1080/2331186X.2022.2034247
41. Kesmodel US. Cross- sectional studies–what are they good for?. Acta obstetricia et gynecologica Scandinavica. 2018; 97(4): 388-393. https://doi.org/10.1111/aogs.13331
42. Wu IC, Pease R, Maker CJ. General education teachers' perceptions of the Real Engagement in Active Problem Solving (REAPS) model. Australasian Journal of Gifted Education. 2021; 30(2): 64-69.
43. Ary D, Jacobs LC, Irvine CKS, Walker DA. Introduction to research in education (10th edition). 172-175. Boston: Cengage; 2019.
44. Opoku, MP, Alsheikh N, Moustafa A, Anwahi N, Aljaberi M, Shah H, Aldarmaki A, Elhoweris H. An Exploration of the Self-Concept of Gifted High School Students in the United Arab Emirates. Current Psychology. 1-22. https://doi.org/10.1007/s12144-023-05393-4
45. Harter S. Manual for the Self-Perception Profile for Children. Denver, CO: University of Denver; 1985.
46. Mengual-Andrés S, Roig-Vila R, Mira JB. Delphi study for the design and validation of a questionnaire about digital competences in higher education. International Journal of Educational Technology in Higher Education. 2016; 13(1), 1-11. https://doi.org/10.1186/s41239-016-0009-y
https://doi.org/10.1080/2331186X.2017.1332840
47. Pallant J. SPSS Survival Manual: A step by step guide to data analysis using IBM SPSS (7th edition). Open Education Press; 2020.

48. Schumacker E, Lomax G. A beginner's guide to structural equation modeling (4th ed.). Routledge; 2016.

49. Tabachnick BG, Fidell LS. Using multivariate statistics (seventh edition). Pearson; 2019.

50. Byrne BM. Structural equation modelling with AMOS: Basic concepts, applications, and programming (third edition). Routledge; 2016.

51. Hayes AF. Introduction to mediation, moderation, and conditional process analysis: a regression-based approach. Third edition. New York; London: The Guilford Press; 2022. 732 p. (Methodology in the social sciences).

The Nexus Between the Attitudes and Self-Concepts of Gifted Students in an Arabian Context

Maxwell Peprah Opoku[1], Ashraf Moustafa[1], Negmeldin Alsheikh[2], Noora Anwahi[1], Mariam Aljaberi[1], Thara Alkhateri[1], Aysha Almeqbaali[1], Hala Elhoweris[1]

[1]Department of Special and Gifted Education, United Arab Emirates University, Al Ain, United Arab Emirates

[2]Curriculum & Methods of Instruction, United Arab Emirates University, Al Ain, United Arab Emirates

Corresponding author: Dr Maxwell Peprah Opoku (Maxwell.p@uaeu.ac.ae), Assistant Professor, Special Education Department, P. O. Box 15551, Al-Ain–United Arab Emirates.

Abstract

Background: The research literature has reported the need for educators to develop suitable programs geared toward the nurturance of gifted students. Although some non-Western countries have adopted policies to foster the growth of exceptional students, their contributions to research in gifted education are limited. To expand the literature on gifted education, this study was guided by Ajzen's theory of planned behavior to explore the attitudes and self-conceptions of gifted and talented students in the United Arab Emirates.

Methods: The revised Self-Perception Profile for Children and Opinions About the Gifted and Their Education scales were used to collect data from 150 high school students in Abu Dhabi, one of seven states (emirates) in the United Arab Emirates. AMOS software version 29 was used to conduct confirmatory factor and path analyses. To evaluate the differences between the background variables on attitudes and self-concepts, t-tests were calculated using SPSS software version 28.

Results: The study found a relationship between the attitudes and self-concepts of students regarding their participation in enrichment programs. The hypothesized moderation effect of demographic variables on the relationship between attitudes and self-concepts was not supported.

Conclusion: The study's limitations and implications for educators in the United Arab Emirates to prioritize programs geared toward developing the attitudes and self-concepts of gifted students are discussed in detail.

Keywords: school, gifted and talented students, gifted education, theory of planned behavior, well-being

Introduction

The need to develop suitable programs for gifted and talented (GT) students has been well explored [1,2]. There is a consensus that GT students can become highly productive citizens who contribute to enriching the communities in which they live by finding solutions to social problems [1,3]. It has been suggested that GT students should be enrolled in appropriate programs that encourage their potential and ability to engage in social problem solving [2,3]. To ensure that GT students derive the maximum benefit from such programs, researchers have paid attention to their self-concepts [4-6] and attitudes toward participating in them [7-10]. Such studies, however, have a relatively limited scope because most have been undertaken in highly developed countries, such as the United States [11,12]. Since GT programs are potentially influenced by the culture and way of life in a given context [13-15], this study aimed to explore the relationship between the attitudes and self-concepts of GT students in a novel Arabian context, the United Arab Emirates (UAE).

The GT concept is not without controversy, and there are two main worldviews regarding giftedness. The first associates giftedness with individuals who excel academically and score at least 140 on IQ testing. This concept of giftedness has been criticized as narrow, restrictive, and favoring a particular group of students from privileged backgrounds [14-16]. Stack et al. [16] argued that defining giftedness according to cognitive ability limits or excludes the enrollment of students from diverse or minority cultural backgrounds in gifted programs. They pointed out that students whose first language is not English are at a disadvantage in enrolling in such programs because the instruments and measurements used to identify GT students are primarily in English. The second worldview is more culture-based and argues that GT is not only related to cognitive ability but also to individuals' ability to manage human relationships [16-18]. Consequently,

there have been calls for the inclusion of informal processes in the concept of GT [13-16]. Even though the GT lens has a limited scope in the UAE and other comparable cultural contexts, there is an ongoing quest for best practices and strategies to promote the development of GT students in these countries [19].

The placement of GT students in programs has been described as labeling [5,11], and there are diverse views concerning ability groupings. For example, one school of thought contends that the identification and subsequent placement of GT students in gifted programs builds their confidence and elicits their best, resulting in improved behavior [11]. The advantages of ability groupings have spurred educators to recognize and place GT students in programs that will boost their development. Conversely, researchers have contended that ability grouping such as enrichment programs is counterproductive as it drives a wedge between those identified as GT and others [5]. As a result, it is challenging for GT students and their peers to form healthy relationships in the same classroom. In addition, some GT students might be complacent because they feel advantaged and unchallenged to work hard to reach their potential [1]. However, despite the risks of identifying and grouping GT students, educators and policymakers should develop initiatives that support them to maximize their potential. GT students will benefit the most from placement in programs that allow their gifts to unfold and fully develop.

Western countries have more experience in developing gifted programs compared with non-Western countries. While the UAE has developed gifted programs for GT students [19], no attempt has been made to understand the attitudes and self-concepts of students enrolled in such programs. The overarching aim of the current study was to investigate the relationship between the attitudes and self-concepts of GT students toward their inclusion in gifted programs.

Theoretical Framework

This study was guided by Ajzen's [20-22] theory of planned behavior (TPB), a major theory used to assess human behavior in societies [23]. The TPB expands on the theory of reasoned action, arguing that behavior is a product or interplay of two related beliefs, behavioral and normative, which impact the intention toward a given behavior [20,24]. Normative beliefs, in contrast to behavioral beliefs, refer to the effect or support of important others on one's intention to perform an action. Behavioral beliefs pertain to one's view of a certain behavior [22]. However, Ajzen [20] later expanded his theory to include a third variable, control beliefs, which might directly impact one's actual behavior. Control beliefs refer to a person's confidence in their capacity to complete a given task or behavior [20,21]. The three related beliefs combine to predict one's intention toward a given behavior. However, although the TPB has been used to guide studies on inclusive education for students with disabilities [22,23], it has rarely been applied to studies of GT students.

For the purpose of this study, the tenets of TPB were adopted. According to Ajzen [20], these tenets develop into variables that predict human behavior. For example, behavioral beliefs develop into attitudes toward a given behavior, normative beliefs develop into subjective norms, and control beliefs develop into perceived behavioral control (used as a proxy for self-concept in this study). In this study, attitudes were operationalized as GT students' perceptions of being categorized and participating in gifted programs. Additionally, subjective norms were explained as the effect or external influence on GT students' involvement in gifted programs. Moreover, self-concept is defined as GT students' understanding of their personalities and confidence in their abilities. According to expectancy-value logic, favorable attitudes toward the three intention components are critical for achieving a desirable outcome [21,22]. Specifically, favorable

attitudes, subjective norms, and positive self-concepts are vital to GT students' success. In other studies on teachers' attitudes toward students with disabilities using the TPB, researchers have reported that favorable attitudes and self-concepts contribute to effective classroom practices [25,26].

Ajzen [20,21] further suggested that the cumulation of subjective norms, attitudes, and self-efficacy explains one's intention to perform a given behavior. The focus of this study, however, was on people's attitudes and self-concepts, because they have direct control over these. Ajzen [20] suggested a relationship between students' attitudes and self-concepts and reasoned that they need to possess favorable attitudes and self-concepts before engaging in a given behavior. In this study, a relationship between attitude and self-concept was proposed. This hypothesis has been tested and supported in studies on inclusive education [25,26].

Ajzen [21] also suggested that the background variables of individuals involved in a given behavior could further explain their intentions. Therefore, human behavior researchers should pay close attention to demographic characteristics and their effects on individual actions or inactions. In this study, it was predicted that background variables could influence the link between attitude and self-concept. In inclusive education research, the TPB has been used to study the association between demographic variables and intentions toward teaching practices [25,26]. However, these studies have yielded contradictory results, and contextual studies of intention are therefore required to develop insights and influence teaching practices. Against this backdrop, in this study, TPB was used as a lens to study the relationship between the attitudes and self-concepts of GT students in the UAE.

Attitudes of Gifted Students

In this study, attitudes refer to students' perceptions of being in a gifted program. According to Gagné [27], understanding attitudes toward gifted education is imperative to develop baseline information about the effectiveness of practices in schools. However, studies on attitudes toward gifted education have been limited mainly to preservice [28] or in-service teachers [12,29]. A large study in Ireland examined teachers' attitudes toward teaching GT students. Although the teachers indicated that they supported gifted education and used specialists to support the teaching of GT students, they had an unfavorable attitude toward grade acceleration. Similarly, in a Swedish study conducted by Ivarsson [30], principals rated their attitudes using the Opinions About the Gifted and Their Education (OGE) scale developed by Gagné and Nadeau [31]. The principals' perceptions of gifted education ranged from negative to ambivalent. However, it is apparent that GT students have not been given many opportunities to share their attitudes toward being in a gifted program.

There is a general consensus that children are "rights bearers" who have the capacity to share or contribute to issues concerning their well-being. Unfortunately, studies on the attitudes of students, such as those in secondary schools, toward their participation in gifted programs are very few. Only a small body of literature has looked at the attitudes of GT students [32] toward gifted programs such as environmental [9,10] and science programs [7,8]. In Turkey, Özarslan [9] conducted studies of GT students participating in a gifted program organized by the Science and Art Center (Bilsem). Both surveys revealed comparable results, demonstrating students' overall positive attitudes toward plants and recycling. However, in the latter study, there was no discernible difference between GT students in terms of age, gender, grade, or years of attendance. In Singapore, studies by Caleon [7] and Lang [8] showed a relationship between

intellectual ability and students' attitudes toward science. Caleon [7] reported that when asked about pursuing science in the future, students with above-average academic abilities and male students were more inclined to respond positively than other groups. Lang [8] discovered that gifted female students had more favorable attitudes toward their interactions with chemistry teachers than male students. Although these findings provide valuable insight into GT students' attitudes toward specific subjects, there are no comparable findings from the UAE.

Previous Studies on GT Students' Self-Concepts

Self-concept has been defined as students' confidence in their abilities and potential. There is a link between student well-being and academic performance [33,34], and underlying this relationship is the self-concept. Researchers have suggested that students with high academic achievement possess positive self-concepts [35]. This finding has highlighted the need for education systems to develop positive self-concepts among students. Consequently, many studies have compared the self-concepts of GT and non-GT students [11,12,30,35-37]. These studies have yielded contradictory findings. Whereas some studies have reported no difference in self-concepts between gifted and non-GT studies [33,36], other studies have shown different results [12, 37]. For instance, in a study conducted across 28 school districts in the United States, McCoach and Rinn [12] indicated a variance between the self-concepts of underachieving and high-achieving students. They reported that GT students scored higher on goal valuation and self-regulation compared to their underachieving counterparts. However, one area of consensus in the research is that gifted and non-gifted students differ in their academic self-concepts [33]. Therefore, to expand the discussion on the importance of self-concept in academic performance, more comparative studies between GT students and non-GT students are needed to inform

educational policy and practice. However, in the UAE, there is no information about the self-concepts of GT students compared with those of their non-gifted counterparts.

Researchers have paid attention to studying the self-concepts of GT students [32]. Many studies have reported differences between students based on their self-concept [4-6,12,33,38,39]. For instance, a review paper by Litster and Roberts [33] compared the differences between GT and non-GT students. On athletics and perceived looks, GT students reportedly scored lower than non-GT students. Gifted students, however, outperformed non-gifted students in both academic performance and perceived behavior. Since the UAE is currently developing its system to encourage gifted education [19], more scholarly attention needs to be paid specifically to students involved in gifted programs beyond comparison studies with students who are not involved in gifted programs.

Overview of the Study

Following the 2008 educational policy in the UAE, the Ministry of Education formulated the country's educational strategy goal of 2010–2020, which was a student-centered approach to education [19,40]. The strategic plan emphasized that every student with an exceptional learning need requires individualized education to enable them to achieve their learning goals. Additionally, the Ministry of Education launched the flagship gifted education enrichment program entitled the "Development of Gifted and Talented Students' Skills." During the 2014 calendar year, the program underwent restructuring and was rebranded the "Integrated System to Identify and Care for Talents." Schools were expected to encourage high achievers to investigate social problems and use innovative problem-solving approaches to solve them [40].

These policy initiatives have contributed to the formation of interest groups and associations supporting the education of GT students across the UAE. Notable is the Hamdan Foundation, which promotes gifted education, teacher development, public awareness, parental training, and the implementation of gifted programs in the UAE [19,40]. Although empirical evidence has revealed discrepancies and inadequate enactment of gifted education in the UAE [19], there is a dearth of empirical studies investigating students' learning experiences in these programs.

Current Study

Based on Ajzen's [20] conception of the TPB, a proposition was made in this study with respect to the relationship between self-concepts and attitudes. According to Gagné [27], the extent to which aspects of attitude predict other variables, such as self-concept, has not received much scholarly attention. In view of this, we hypothesized the following:

Hypothesis I: GT students' self-concepts will predict their attitudes toward their involvement in gifted programs (see Figure 1).

Additionally, demographic variables have been identified as vital in efforts to understand the intentions toward a given behavior. For example, in the UAE, gender is a major factor in the grouping of secondary schools according to their high-ability areas, and, typically, secondary school students attend single-sex classrooms. Consequently, their enrollment in gifted programs has followed a gendered pattern. In this study, gender and high-ability area were selected as variables of interest to explore their impact on the relationship between students' self-concepts and their attitudes toward gifted programs:

Hypothesis II: Demographic variables will be a significant moderator of the attitudes and self-concepts of gifted students.

The available evidence has indicated that GT students' attitudes and self-concepts have been studied individually. However, there is little or no empirical research on the relationship between GT students' attitudes and self-concepts. Studies examining students' attitudes and self-concepts are also sporadic in Arabian contexts. Therefore, there is a need to examine the relationship between attitudes and self-perceptions among GT students in the UAE. This study aimed to answer the following research questions:

1. What are the differences between GT students in demographic variables, attitudes, and self-concepts?
2. Do GT students' self-concepts predict their attitudes toward participating in gifted programs in the UAE?
3. Do demographic variables moderate the self-concepts and attitudes of GT students toward participating in gifted programs in the UAE?

Methods

Design

The study entailed cross-sectional, descriptive research that aimed to capture the self-concepts of students at a given time [41]. The design was appropriate as it enabled the study of students' attitudes and self-concepts at a given point in time.

The participants in this study were GT high school students nominated by their schools to participate in an enrichment program. In the UAE, there is a three-tier educational structure:

cycle one (pre-school to grade 4), cycle two (grades 5–8), and cycle three (grades 9–12). While public schools are managed by the Emirates Schools Establishment, they are also supervised by the Abu Dhabi Department of Education and Knowledge (ADEK). In this study, GT students were identified based on their performance on IQ tests conducted in the schools. GT students participate in projects related to the science, technology, engineering and mathematics (STEM) areas of high ability. For instance, some of the students have been involved in the Maker's Real Engagement in Active Problem-Solving project [42] on environmental pollution.

Table 1. Difference between background variables, attitudes, and self-concept

	Sample	Attitude	Self-concept
**Gender**			
Male	37 (25%)	3.62 (.54)	3.77 (.65)
Female	113 (75%)	3.66 (.55)	3.66 (.61)
t		-.34	.91
Cohen's d		.06	.17
**Age**			
10–15 years	38 (25%)	3.62 (.56)	3.66 (.57)
16–18 years	112 (75%)	3.66 (.54)	3.69 (.64)
t		-.33	-.27
Cohen's d		.06	.05
**Grade Level**			
Intermediary level (Grades 7–9)	40 (27%)	3.65 (.65)	3.75 (.66)
High School (Grades 10–12)	108 (73%)	3.65 (.51)	3.66 (.61)
t		.009	.08
Cohen's d		.002	.15
**Area**			
STEM areas	110 (73%)	3.64 (.55)	3.69 (.66)
Other areas	40 (27%)	3.68 (.54)	3.67 (.51)
t		-.37	.15
Cohen's d		.07	.03

STEM: science, technology, engineering and mathematics

Simple random sampling was used to recruit the study participants from the sampling frame [43]. Each student involved in the enrichment program was invited to participate in this

study. The current study drew GT students from private schools, as they were the main schools running enrichment programs for GT students. The decision to include students from private schools was based on ADEK granting its permission to include students on their high-ability roster. Prior consent was sought from all parents whose children were on the ADEK register. Invitations were sent via email to all GT students participating in the enrichment program. A total of 150 students took part (see Table 1).

Data Collection

This study forms part of a larger project that sought to understand the well-being of GT students participating in an enrichment program in the UAE [44]. The study was approved by the Social Sciences Ethics Review Committee of the United Arab Emirates University (ERS_2021_8436).

The survey was made up of three sections. The first section collected demographic data (gender, age, grade level, and high-ability area(s)), which were included based on a review of the previous literature [8-10,12].

The ensuing parts of the instrument were published scales related to the components (attitudes and self-concepts used as a proxy for self-efficacy) of Ajzen's [20] TPB, which was adapted for this study. Specifically, the second part [45] used a revised Self-Perception Profile for Children (SPPC) scale, which has been used extensively to study the self-concept.

The scale is made up of 34 items scored on a 5-point scale: strongly disagree (1) to strongly agree (5). The scale comprises six subscales (scholastic competence [students' rating of their academic ability], *n* = 5; social acceptance [ability of students to develop healthy social relationships], *n* = 6; athletic competence [competence of students in extracurricular sporting

activities], $n = 6$; physical appearance [extent to which students accept themselves], $n = 5$; behavioral conduct [students' ratings of their emotional regulations], $n = 6$; and global self-worth [defined as how students appreciate and embrace themselves], $n = 6$).

The third part was a revised 20-item OGE scale [31]. The OGE scale was adopted for this study because it has been used extensively to study teachers' attitudes [27,32]. However, in Gagné's [27] view, the instrument needed to be more efficient and concise to capture the core components of attitudes. Based on feedback from expert reviewers, the authors settled on the revised instrument, which is short and captures the important aspects of attitude that can be reported by secondary school students. Additionally, this appears to be the first time the OGE has been implemented for students in a non-Western context; thus, we needed to use a version that students could easily comprehend. These justifications informed the use of the abridged version of the original instrument. Responses to the measure, which consists of three subscales (support, acceleration, and elitism), are anchored on a 5-point Likert scale ranging from strongly disagree (1) to strongly agree (5).

The instrument consisted of three subscales. First, support for gifted education is comprised of 10 items and refers to the extent to which participants agree with their involvement in a special program for the gifted. Elitism, which comprises six items, assesses participants' beliefs as to whether being involved in a gifted program represents favorable or preferential treatment given to gifted students. Acceleration, constituting four items, measures beliefs about the acceleration of gifted students in schools [12].

Three specialists were given the questionnaire to assess and remark on its suitability for data gathering. This process is called the Delphi approach [46], which is a review of instruments by experts with in-depth knowledge who evaluate the suitability of a questionnaire before its

implementation. Two versions of the instrument were given to the experts so that they could determine the version most suitable for data collection. There was consensus among the specialists on the use of the short version for data collection for this study. Following this, the instrument was piloted with eight GT students who were not considered for participation to determine whether it was clear and simple to understand.

An official invitation was sent to ADEK seeking permission to conduct this study. The parents of children on the high-ability roster gave ADEK their consent before their email contacts were released to the research team. The parents were informed of the study's goals and provided a breakdown of its methodology. They were also assured that no identifiable information about their children would be used or shared with anyone outside the research team. The parents were further informed that their children would not be incentivized to participate in this study. The children whose parents responded positively provided informed consent for participation in this study.

Google Forms was employed for data collection due to the mobility challenges imposed by the COVID-19 pandemic. There were Arabic and English translations of the items on the instrument. The data were collected from May 2022 to July 2022. The duration of the survey completion was 15–25 minutes.

Data Analysis

The data were cleaned in Microsoft Excel before being imported to the Statistics Package for Social Science (SPSS) version 28 for analysis. The initial computation of missing data using missing at-random tests showed a range of 0.6% to 1.7%. Following this, the missing data were imputed. After that, a normality test was performed to determine whether the data were

appropriate for parametric analysis. The results of the Kolmogorov–Smirnov and Shapiro–Wilk tests were insignificant, suggesting the normality of the data for parametric testing [47].

While the SPPC was validated in an earlier study [44], the OGE was used for the first time in the current context and thus, tt was necessary to assess its construct validity. Consequently, confirmatory factor analysis (CFA) was computed to understand OGE's dimensionality. The following goodness-of-fit indices were used: a chi-square value of less than 0.5, a comparative fit index (CFI) and a Tucker-Lewis index (TLI) of 0.90 or greater, a root square error of approximation (RMSEA), a standard root mean square residual (SRMR) of at least 0.08, and a regression weight of at least 0.50 [48,49]. However, if the model did not meet the above thresholds, modification indices were observed to eliminate items with high and erroneous covariances [50].

The computation CFA for the 20-item OGE scale showed a poorly fit model: chi-square = 3.02, CFI = 0.60, TLI = 0.53, RMSEA = .012, and SRMR = 0.11. The items on the elitism subscale had poor loadings (items 11, 12, 13, 14, 15, and 16), and thus were deleted from the model. Additionally, half of the items of the support subscale (5, 7, 8, 9, and 10) had factor loadings below the cutoff and were removed from the model (see Figure 1). The rerun reached acceptable indices: chi-square = 1.12, CFI = 0.99, TLI = 0.98, RMSEA = 0.03, and SRMR = 0.05. However, there was no correlation between the subscales support and acceleration ($r = -.08$).

The instrument's reliability was assessed using Cronbach's alpha: attitude (0.60) and self-concept (0.90). The outcomes confirmed that the tool was suitable for reporting the study.

Figure 1. Confirmatory factor analysis for an opinion about the gifted and their education

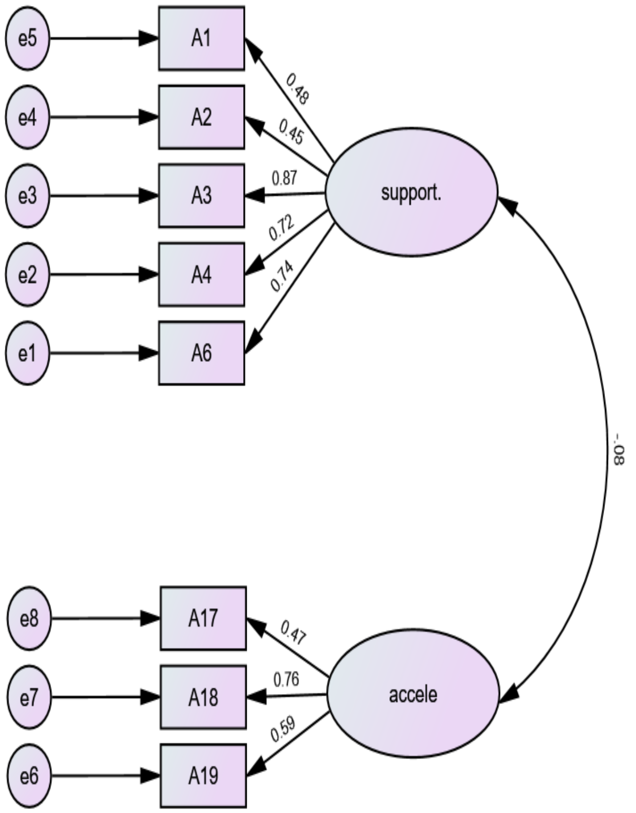

Note: accele = acceleration; support = support for gifted programs

For Research Question 1, which concerned the effects of GT students' demographics on attitudes and self-concepts, t-tests were used because all the demographics had two levels. An assumption of variance homogeneity was observed to ensure that it was not violated [47].

To answer Research Question 2, direct path analysis, a form of structural equation modeling, was used to test the relationship between attitudes and self-concepts. Path analysis

was used because it is appropriate for testing hypotheses [50], such as the assumed relationship between attitude and self-concept.

A preliminary CFA was used to determine the relationship between overall attitudes and self-perceptions, and the results were interpreted using the following thresholds: small (0.10–0.30), moderate (0.31–0.50), and large (≥ 0.51) [47]. The contribution of self-concept to the variance in attitudes was computed using path analysis. Based on Gagné's [27] suggestion that researchers investigate the types of attitudes and the specific behaviors they influence, path analysis was conducted at the subscale levels. To ensure that the model was appropriate, the fit indices stated above were observed.

For Research Question 3, the two demographic variables of interest—gender and students' ability areas—were added to the earlier path analysis model to assess their interaction effects on the association between attitudes and self-concepts. Both bootstrap and bias-corrected confidence intervals were set at 500 and 95%, respectively. In cases where there was a significant moderation effect, Andrew Hayes's [51] PROCESS model was used to explore the direct effect of the moderators on attitudes and self-concepts.

Results

Association Between Demographics, Attitude, and Self-Concept

The computation of mean scores showed students' neutrality on attitudes (*M* = 3.65; *SD* = 0.54) and self-concepts (*M* = 3.69; *SD* = 0.62). Additionally, the mean scores for self-concept showed that participants were high on global self-worth (*M* = 4.12; *SD* = 0.91) but ambivalent on the remaining subscales (scholastic competence, *M* = 3.94, *SD* = 0.74; social acceptance, *M* =

3.49, *SD* = 1.20; athletic competence, *M* = 3.01, *SD* = 1.11; physical appearance, *M* = 3.42, *SD* = 1.29; behavioral conduct, *M* = 3.93, *SD* = 1.29; see Table 2).

Table 2. Summary of mean scores on self-concept

	**Items**	M	SD
	**Scholastic competence**	3.94	.91
W1	I am good at schoolwork	4.34	.82
W3	I remember lesson easily	3.77	.94
W4	I perform very well in science and mathematics	3.92	.94
W5	I figure out answer easily	3.72	.93
	**Social acceptance**	3.49	1.20
W7	I have a lot of friends	3.71	1.24
W9	I do things with a lot of friends	3.64	1.15
W11	I am very popular in school	3.12	1.21
	**Athletic competence**	3.01	1.11
W12	I am very good at sports	3.32	1.10
W15	I am better than others in sports	2.79	1.15
W17	I am good at both outdoor and indoor sports	2.92	1.07
	**Physical appearance**	3.42	1.29
W18	I am very happy with my physical appearance	3.48	1.22
W19	I am happy with my height and weight	3.31	1.34
W20	I like my body as it is	3.46	1.27
W21	I like my physical appearance as it is	3.42	1.32
	**Behavior conduct**	3.93	1.29
W23	I like my behaviour	4.13	.94
W24	I usually do the right thing	3.77	1.00
W25	I usually do things in the right way	3.83	.89
W28	I behave well all things	3.98	.97
	**Global self-worth**	4.12	.97
W29	I am happy with myself	4.12	.96
W31	I am happy with myself as a person	4.12	.95
W32	I like myself	4.17	.97
W33	I am happy the way I am	4.05	1.01

Concerning the subscale on attitudes, the participants were ambivalent about support (*M* = 3.71, *SD* = 1.02) and acceleration (*M* = 3.56, *SD* = 1.10; see Table 3).

Table 3. Summary of mean scores on attitude towards gifted education

	**Item**	**M**	**SD**
	**Support**	3.71	1.02
A1	I have happy being in a gifted program in my school.	4.11	.91
A2	Most of my family and friends consider me gifted.	2.89	1.33
A3	I consider myself to be gifted	3.91	.94
A4	Most of my family and friends are gifted.	3.97	.94
A6	Our schools offer appropriate special education services for the gifted.	3.65	1.00
	**Acceleration**	3.56	1.10
A17	Most gifted children who skip a grade have difficulties in their social adjustment to a group of older students.	3.69	1.21
A18	Children who skip a grade are usually pressured to do so by their parents.	3.58	.97
A19	When skipping a grade, gifted students miss important ideas	3.93	1.13

To determine whether there was an association between background variables, attitudes, and self-concepts, independent t-tests were conducted. The outcomes revealed no differences in the participants' attitudes or self-perceptions (see Table 1).

Predictors of Attitudes

Path analysis was computed to ascertain whether self-concepts could contribute to the variance in attitudes (see Figure 2). An initial CFA showed a significant correlation between overall attitudes and self-concepts (r = 0.81).

Figure 2. Contribution of self-conception in the variance in attitude

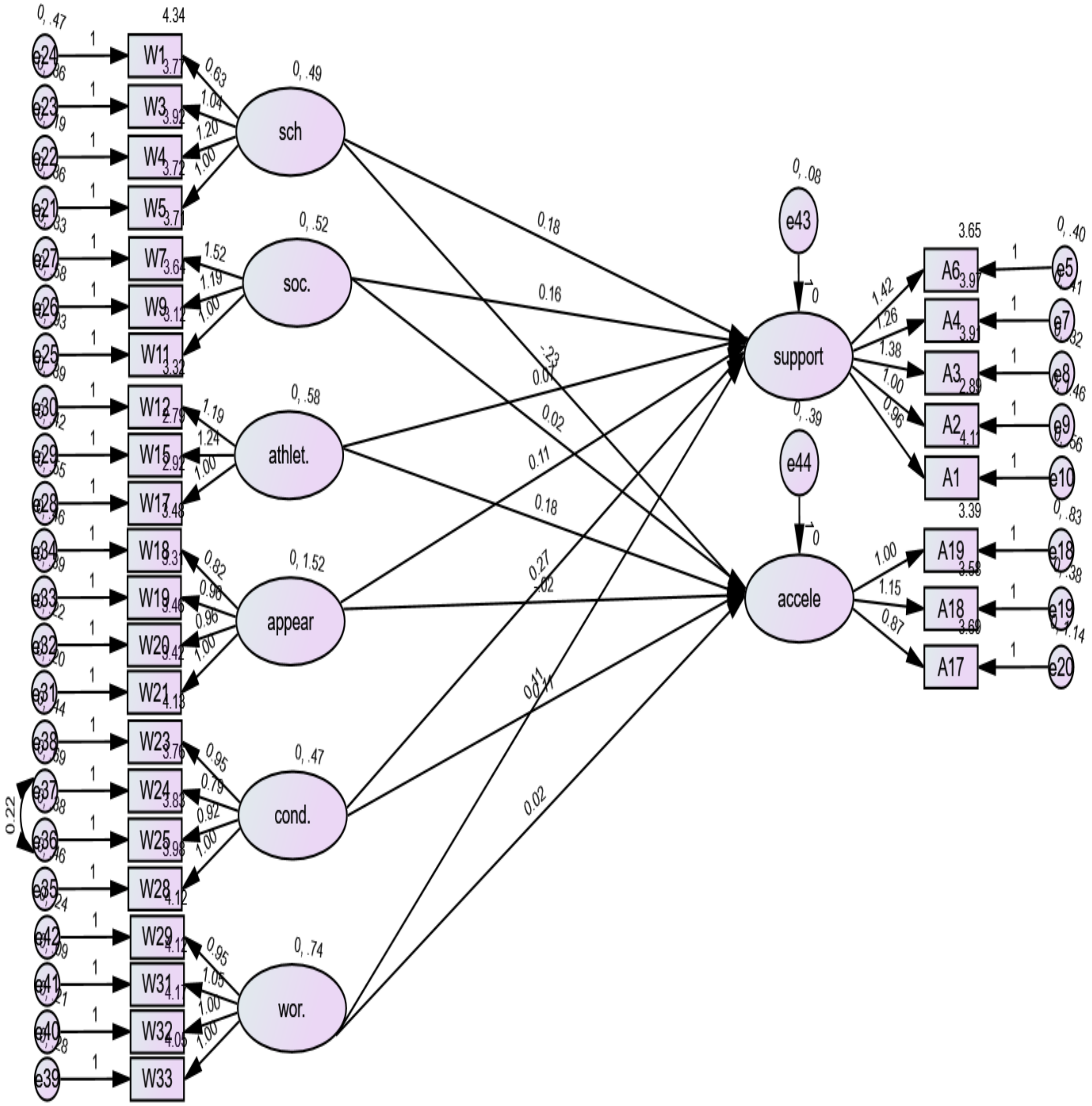

Note: sch = scholastic competence; soc. = social acceptance; athlete. = athletic competence; appear = physical appearance; cond. = behavior conduct; and wor. = global self-worth).

The contribution of self-concept to the variance in attitudes was assessed. First, regarding the attitude subscale of support, the following subscales of self-concept significantly contributed to its variance: scholastic competence (b = 0.30, p = 0.01), social competence (b = 0.28, p = 0.02), physical appearance (b = 0.33, p = .005), behavioral conduct (b = 0.45, p = .004), and global self-worth (b = 0.23, p = .02). Second, concerning the attitude subscale of acceleration, only one predictor made a significant contribution to the variance: scholastic competence (b = −0.24, p =.03).

Moderation

To comprehend the impact of gender and ability area on the relationship between attitude and well-being, moderation analyses were performed. First, gender was operationalized as a moderator to understand its influence on the relationship between acceleration and self-concept. The initial model looked at how gender affected one subscale of attitude and self-concept. Gender had a direct effect only on physical appearance (b = .03, p =. 04). However, gender did not influence acceleration (b = −.24, p =.52). Concerning the standardized indirect effect, the results showed that gender had no interaction effect on the relationship between acceleration and self-concept.

Second, the moderation effect of gender on the relationship between the second subscale of attitude, support, and well-being was assessed. Regarding the standardized direct effect, gender had no direct influence on support (b = −.10, p = .55). Furthermore, the association between support for gifted education and well-being was unaffected by gender.

The interaction effect of a high-ability area on the link between attitude and well-being was also assessed. For instance, the area of high ability had a standardized direct effect on social

competence (b = .08, p = .01) and scholastic competence (b = −.60, p = .01). Additionally, the area of high ability had no direct influence on acceleration (b = −.13, p = .61) and support for gifted education (b = −.22, p = .63). Areas of high ability did not have a standardized indirect effect on the association between attitude and self-concept. Similar findings were observed regarding the degree to which grade level and age moderated the link between attitudes and self-perceptions.

Discussion

In this study, Ajzen's [20] TPB was adopted to understand the relationship between the attitudes and self-concepts of GT students in a novel cultural context, the UAE. The CFA used in the study, whose factorial validity was supported, was used to test the structural validity of the OGE and SPPC scales. The results showed a six-factor structure for the SPPC and a two-factor structure for the OGE. Although modifications were deployed to eliminate reduced items whose erroneous covariances affected the model fit indices [50], the tools were validated in a context other than the usual Western context, where such studies are usually conducted. The validation process involved the observation of numerous parameters (chi-square, CFI, TLI, modifications, etc.) [5,48,50] before ascertaining their validity. The results demonstrate the potential of the SPPC and OGE measures to examine talented students' attitudes and self-perceptions in the UAE and other Arabian countries.

The results of the study supported Hypothesis I and demonstrated a relationship between self-concept and attitude. This finding supports Ajzen's [20] proposition of a relationship between individuals' attitudes and self-concepts in explaining their intention toward a given

behavior. The results are consistent with earlier studies on inclusive education, which revealed a favorable association between people's attitudes and perceptions of their own actions [25,26]. The findings suggest that in the UAE context, as the attitudes of GT students toward participation in gifted programs improve, the students' self-concepts may also improve. The UAE government's top priority is to educate and prepare future generations to support the nation's development [40]. However, achieving this goal relies heavily on GT students, who must be identified and placed in requisite programs where their skills will be nurtured. Unquestionably, if educators make conscious efforts to improve students' attitudes, which might affect GT students' self-perceptions, and vice versa, such a goal could be accomplished.

Although Ajzen [20] argued about the potential for demographic variables to impact intention toward a given behavior, this position was not supported in this study. Background factors were originally thought to have an impact on how GT students in the UAE viewed themselves with respect to their attitudes. However, the findings showed that no demographic variables had an association with or moderated the relationship between attitude and self-concept. This conclusion conflicts with earlier GT studies that found that attitudes and self-perceptions were influenced by demographic factors [6-8]. The study finding is also inconsistent with previous inclusive education research, which reported the influence of demographic variables on attitudes and self-concepts toward learning [25,26]. In the current study, the participants were from private schools only, which could influence the trend identified in this study. Therefore, future studies could examine the attitudes and self-perceptions of GT students in both public and private schools.

One curious finding was the lack of a relationship between the two attitude subscales, support and acceleration. Attitudes explain the disposition of participants toward being identified

and included in gifted programs. Ajzen [20] contended that belief accumulation explains attitudes toward a given behavior, such as being involved in gifted programs. However, in this study, the findings showed no relationship between acceleration and support for gifted programs. Notably, the study's participants engaged in enrichment activities that could have influenced their results. There is a possibility that they were aware of the enrichment program and not the acceleration program. Anecdotal data indicate that many schools in the UAE discourage acceleration, particularly in private institutions. However, the findings indicated that the participants were unsure of support for gifted programs. The UAE government is currently looking at programs to enhance GT students' development [19,40]. This could trigger or expedite discussions among UAE educators on the best methods and support programs for GT students in the country.

The results appear to support the multidimensionality of self-concept in the context of the current investigation. According to Winne et al. [34], self-concept should be studied with a multiple lens to develop a holistic understanding. For example, the effectiveness of studies may be impacted by both classroom procedures and extracurricular activities. The association between the self-concept subscales seems to provide useful guidance for education practices in the UAE, even if the purpose here is not to establish causation. It is useful for educators in the UAE to observe a multiplicity of factors that could negatively impact the confidence of GT students. Indeed, a significant correlation was observed between self-worth and behavior management. The tendency of GT students to exhibit unfavorable behavior has been mentioned in some literature, which might affect how well they succeed [1,2]. In the UAE context, to avert this situation, policymakers might consider developing the self-worth of individuals by building their self-confidence and beliefs, which could positively affect their behavioral conduct.

Conclusion

The study was guided by Ajzen's [20] TPB. The main objective was to investigate the connection between GT students' attitudes and self-perceptions in the UAE context. The TPB [20] makes two important propositions: (a) there is a relationship between the predictors and intentions toward a given behavior, and (b) background variables have an influence on the predictors. In the research on GT students in the UAE, these two hypotheses were put to the test. One proposition was supported by the study's findings, whereas the other was not. The study's findings showed a substantial relationship between GT students' self-concepts and attitudes. Conversely, demographic variables did not influence the relationship between attitudes and self-concepts. In conclusion, the study supports the idea that attitudes and self-concepts are crucial in creating a successful program for GT students in the UAE and other similar settings.

Limitations and Recommendations for Future Research

It is impossible to generalize our findings because the study was constrained in certain ways. First, the researchers recruited GT students from private schools participating in an enrichment program in one of the seven states in the UAE. Hence, the interpretation of the study's findings is limited to students from private schools participating in an enrichment program. However, the UAE is a diverse society with students from varied backgrounds and cultures enrolled in private schools. Moreover, one regulating agency, the Ministry of Education, oversees and approves teaching standards and maintains schools. Although the findings could reflect the situation more generally in the country, future research could extend this study to public schools to compare the attitudes and self-concepts of GT students enrolled in both private

and public schools. Second, the data were collected online, and the opportunity to ask the students questions for clarification may have been limited. Future research could use qualitative techniques to develop thorough insights into the experiences of GT students. Third, the findings are an account of GT students only, and it is uncertain whether non-GT students could share the same level of attitudes and self-concepts. Future research could examine how GT and non-GT students view themselves to develop holistic insights into their self-concepts and attitudes toward gifted education.

Nevertheless, the findings could have implications for policymaking in the UAE and other similar cultural contexts. First, policymakers could consider developing awareness and training programs for GT students geared toward enabling them to understand their contributions to national development. Additionally, GT students could be made more aware of why it is important for them to participate in gifted programs. Furthermore, GT students could be involved in extracurricular activities to help improve their self-concepts. For instance, aside from academic programs, educators could consider organizing athletic programs and related activities for GT students to improve their self-concepts. Third, educators could consider clarifying the scope of gifted programs in the UAE. This could include the criteria for acceleration and fine-tuning acceleration programs, which are conterminous with contextual realities. The UAE is working to enhance students’ well-being, and measures such as those suggested above could assist in fostering an environment that is favorable to the development of GT students.

List of abbreviations

CFA	Confirmatory factor analysis
CFI	Comparative fit index
EFA	Exploratory factor analysis
GT	Gifted and talented
SRMR	Standard root mean square residual
UAE	United Arab Emirates

Declarations

Availability of data statement

The datasets generated and analysed during the current study are not publicly available due to ethical restrictions but are available from the corresponding author upon reasonable request.

Financial disclosure

The study was funded by the Office of the Associate Provost for Research at United Arab Emirates University. The funders had no role in study design, data collection and analysis, decision to publish, or preparation of the manuscript.

Conflict of interest statement

The authors declare that they have no competing interests.

Acknowledgements

We would like to thank the Abu Dhabi Department Knowledge and Education for their support during recruitment and data processing. Our heartfelt gratitude goes to all students and parents who participated in this study.

Authors' contributions

Conceptualisation: Maxwell Peprah Opoku, Ashraf Moustafa, Negmeldin Alsheikh, Noora Anwahi, Mariam Aljaberi, Thara Alkhateri, Aysha Almeqbaali, Hala Elhoweris
Data curation: Maxwell Peprah Opoku, Ashraf Mustafa
Formal analysis: Maxwell Peprah Opoku
Investigation: Maxwell Peprah Opoku,
Methodology: Maxwell Peprah Opoku, Ashraf Moustafa, Negmeldin Alsheikh, Noora Anwahi, Mariam Aljaberi, Thara Alkhateri, Aysha Almeqbaali, Hala Elhoweris
Project administration: Maxwell Peprah Opoku
Resources: Maxwell Peprah Opoku
Writing – original draft: Maxwell Peprah Opoku, Ashraf Moustafa, Negmeldin Alsheikh, Noora Anwahi, Mariam Aljaberi, Thara Alkhateri, Aysha Almeqbaali, Hala Elhoweris
Writing and reviewing the draft: Maxwell Peprah Opoku, Ashraf Moustafa, Negmeldin Alsheikh, Noora Anwahi, Mariam Aljaberi, Thara Alkhateri, Aysha Almeqbaali, Hala Elhoweris

References

1. Heward WL, Ford DY. Gifted and Talented. In William L. Heward Exceptional children: An introduction to Special Education (10th Edition). Pearson; 2013.

2. Merrotsy P. Supporting outstanding learners. In Adrian Ashman (editor), Education for Inclusion and diversity (p. 233-264). Pearson; 2015.
3. Kuo CC, Maker J, Su FL, Hu, C. Identifying young gifted children and Cultivating problem solving abilities and multiple intelligences. Learning and Individual Differences. 2010; 20(4): 365–379.
4. Cross TL, Swiatek MA. Social coping among academically gifted adolescents in a residential setting: A longitudinal study. Gifted Child Quarterly. 2009; 53(1): 25-33. 10.1177/0016986208326554
5. Hoge RD, Renzulli JS. Exploring the link between giftedness and self-concept. Review of Educational Research. 1993; 63(4): 449-465. https://doi.org/10.3102/00346543063004449
6. Košir K, Horvat M, Aram U, Jurinec N. Is being gifted always an advantage? Peer relations and self-concept of gifted students. High Ability Studies. 2016; 27(2), 129–148. https://doi.org/10.1080/13598139.2015.1108186
7. Caleon IS, Subramaniam R. Attitudes towards science of intellectually gifted and mainstream upper primary students in Singapore. Journal of Research in Science Teaching: The Official Journal of the National Association for Research in Science Teaching. 2008; 45(8): 940-954. https://doi.org/10.1002/tea.20250
8. Lang QC, Wong AFL, Fraser Barry J. Teacher-Student Interaction and Gifted Students' Attitudes toward Chemistry in Laboratory Classrooms in Singapore. The Journal of Classroom Interaction. 2005; 40(1): 18-28

9. Özarslan M. Perceptions and attitudes of gifted and talented secondary school students towards plants. Gifted Education International. 2020; 36(3); 266-284. https://doi.org/10.1177/0261429420946735
10. Ugulu I. A quantitative investigation on recycling attitudes of gifted/talented students. Biotechnology & biotechnological equipment. 2015; 29(sup1): S20-S26. https://doi.org/10.1080/13102818.2015.1047168
11. Berlin JE. It's all a matter of perspective: Student perceptions on the impact of being labeled gifted and talented. Roeper Review. 2009; 31(4): 217-223. https://doi.org/10.1080/02783190903177580
12. McCoach DB, Rinn D. Factors that differentiate underachieving gifted students from high-achieving gifted students. Gifted Child Quarterly. 2007; 47(2): 144-154. https://doi.org/10.1177/001698620304700205
13. Callahan CM. Identifying gifted students from underrepresented populations. Theory into Practice. 2005; 44(2): 98-104, https://doi.org/10.1207/s15430421tip4402_4
14. Ford DY. Segregation and the Underrepresentation of Blacks and Hispanics in Gifted Education: Social Inequality and Deficit Paradigms. Roeper Review. 2014; 36(3): pp. 143–154, DOI: 10.1080/02783193.2014.919563
15. Milner HR, Ford DY. Cultural considerations in the underrepresentation of culturally diverse elementary students in gifted education. Roeper Review. 2007; 29(3): 166-173. https://doi.org/10.1080/02783190709554405
16. Stack N, Sutherland M, Smith TA, Tungaraza FD. Creating a space for diverse learners in multifarious contexts. In Taber KS, Sumida, M. International perspectives on Science

education for the gifted: key issues and challenges (pp. 140–153). Taylor and Francis; 2016.

17. Maree JG. Gifted education in Africa. In S. I. Pfeiffer, E. Shaunessy-Dedrick, & M. Foley-Nicpon (Eds.), APA handbook of giftedness and talent (pp. 131–142). American Psychological Association; 2018. https://doi.org/10.1037/0000038-009
18. Ngara C. Gifted education in Zimbabwe. Cogent Education. 2017; 4(1): 1–14. https://doi.org/10.1080/2331186X.2017.1332840
19. AlGhawi MA. Gifted education in the United Arab Emirates. Cogent Education. 2017; 4(1): 1368891. https://doi.org/10.1080/2331186X.2022.2034247
20. Ajzen I. The theory of planned behavior. Organizational Behavior and Human Decision Processes. 1991; 50(2): 179–211. https://doi.org/10.1016/0749-5978(91)90020-T
21. Ajzen I. The theory of planned behaviour: reactions and reflections. Psychology & Health. 2011; 26(9): 1113–1127. https://doi.org/10.1080/08870446.2011.613995
22. Ajzen I. The theory of planned behavior: Frequently asked questions. Human behavior and emerging technologies. 2020; 2(4): 314-324. https://doi.org/10.1002/hbe2.195
23. Cheng EW. Choosing between the theory of planned behavior (TPB) and the technology acceptance model (TAM). Educational Technology Research and Development. 2019; 67: 21-37. https://doi.org/10.1007/s11423-018-9598-6
24. La Barbera F, Ajzen I. Control interactions in the theory of planned behavior: Rethinking the role of subjective norm. Europe's Journal of Psychology. 2020; 16(3); 401. https://doi.org/10.5964/ejop.v16i3.2056
25. Opoku MP, Cuskelly M, Pedersen SJ, Rayner SC. Attitudes and Self-Efficacy as Significant Predictor of Intention of Secondary School Teachers towards the

Implementation of Inclusive Education in Ghana. European Journal of Psychology of Education. 2021; 36: 673-691. https://doi.org/10.1007/s10212-020-00490-5

26. Opoku MP, Cuskelly M, Pedersen SJ, Rayner SC. Applications of the Theory of Planned Behaviour in assessing intentions of teachers towards practicing inclusive education: a scoping review. European Journal of Special Needs Education. 2021; 36(4): 577-592. https://doi.org/10.1080/08856257.2020.1779979

27. Gagné F. Attitudes toward gifted education: Retrospective and prospective update. Psychological Test and Assessment Modeling. 2018; 60(4): 403. https://doi.org/10.1080/02783193.2007.11869221

28. Camcı-Erdogan S. Investigating pre-service gifted education teachers' self-efficacy toward science teaching and scientific attitudes. Eurasian Journal of Educational Research. 2015; 59: 133-148. http://dx.doi.org/10.14689/ejer.2015.59.8

29. Cross TL, Cross JR, O'Reilly C. Attitudes about gifted education among Irish educators. High Ability Studies. 2018; 29(2): 169-189. https://doi.org/10.1080/13598139.2018.1518775

30. Ivarsson L. Principals' perceptions of gifted students and their education. Social Sciences & Humanities Open. 2023; 7(1): 100400. https://doi.org/10.1016/j.ssaho.2023.100400

31. Gagné F, Nadeau L. Opinions about the gifted and their education. Unpublished instrument; 1991.

32. Cross JR, Cross T, Frazier AD. Student and teacher attitudes toward giftedness in a two-laboratory school environment: A case for conducting a needs assessment. NALS Journal. 2013; 5(1): 1-21.

33. Litster K, Roberts J. The self-concepts and perceived competencies of gifted and non-gifted students: A meta-analysis. Journal of Research in Special Educational Needs. 2011; 11: 130–140. doi:10.1111/j.1471-3802.2010.01166.x

34. Winne PH, Woodlands MJ, Wong BY. Comparability of self-concept among learning disabled, normal, and gifted students. Journal of Learning Disabilities. 1982; 15(8): 470–475. https://doi.org/10.1177/002221948201500805

35. Yan K, Haihui Z. A Decade Comparison: Self-Concept of Gifted and Non-Gifted Adolescents. International Education Journal. 2005; 6(2): 224-231.

36. Peperkorn C, Wegner C. Academic self-concept of gifted and nongifted biology students: indications for domain-specific gifted education. Progress in Science Education (PriSE). 2023; 6(2): 6-19. https://doi.org/10.25321/prise.2023.1326

37. Preckel F, Schmidt I, Stumpf E, Motschenbacher M, Vogl K, Scherrer V, Schneider W. High- ability grouping: Benefits for gifted students' achievement development without costs in academic self- concept. Child development. 2019; 90(4): 1185-1201. https://doi.org/10.1111/cdev.12996

38. Rinn AN, Mendaglio S, Moritz Rudasill K, McQueen KS. Examining the relationship between the overexcitabilities and self-concepts of gifted adolescents via multivariate cluster analysis. Gifted Child Quarterly. 2010; 54(1): 3-17. https://doi.org/10.1177/0016986209352682

39. Rudasill KM, Callahan CM. Psychometric characteristics of the Harter Self-Perception Profiles for adolescents and children for use with gifted populations. Gifted Child Quarterly. 2008; 52(1): 70-86. https://doi.org/10.1177/001698620731105

40. Ismail SA, Alghawi MA, AlSuwaidi KA. Gifted education in United Arab Emirates: Analyses from a learning-resource perspective. Cogent Education. 2022; 9(1): 2034247. https://doi.org/10.1080/2331186X.2022.2034247

41. Kesmodel US. Cross- sectional studies–what are they good for?. Acta obstetricia et gynecologica Scandinavica. 2018; 97(4): 388-393. https://doi.org/10.1111/aogs.13331

42. Wu IC, Pease R, Maker CJ. General education teachers' perceptions of the Real Engagement in Active Problem Solving (REAPS) model. Australasian Journal of Gifted Education. 2021; 30(2): 64-69.

43. Ary D, Jacobs LC, Irvine CKS, Walker DA. Introduction to research in education (10th edition). 172-175. Boston: Cengage; 2019.

44. Opoku, MP, Alsheikh N, Moustafa A, Anwahi N, Aljaberi M, Shah H, Aldarmaki A, Elhoweris H. An Exploration of the Self-Concept of Gifted High School Students in the United Arab Emirates. Current Psychology. 1-22. https://doi.org/10.1007/s12144-023-05393-4

45. Harter S. Manual for the Self-Perception Profile for Children. Denver, CO: University of Denver; 1985.

46. Mengual-Andrés S, Roig-Vila R, Mira JB. Delphi study for the design and validation of a questionnaire about digital competences in higher education. International Journal of Educational Technology in Higher Education. 2016; 13(1), 1-11. https://doi.org/10.1186/s41239-016-0009-y https://doi.org/10.1080/2331186X.2017.1332840

47. Pallant J. SPSS Survival Manual: A step by step guide to data analysis using IBM SPSS (7th edition). Open Education Press; 2020.

48. Schumacker E, Lomax G. A beginner’s guide to structural equation modeling (4th ed.). Routledge; 2016.
49. Tabachnick BG, Fidell LS. Using multivariate statistics (seventh edition). Pearson; 2019.
50. Byrne BM. Structural equation modelling with AMOS: Basic concepts, applications, and programming (third edition). Routledge; 2016.
51. Hayes AF. Introduction to mediation, moderation, and conditional process analysis: a regression-based approach. Third edition. New York; London: The Guilford Press; 2022. 732 p. (Methodology in the social sciences).

Dear editor,

Thanks for your feedback on our submission. As you can see, the paper has been edited for clarity. More so, we have taken time to incorporate feedback from the reviewers. Below is our response to your feedback on our revised resubmission.

Comments	Response	Page
**Reviewer 1**		
I highly recommend mentioning the selection method related to the sampling and citing it with relevant literature. The current study drew on Gifted students from private schools as they were running enrichment program for GT students. The decision to include students in private schools was based on the fact that permission ADEK granted permission to include students on their high-ability roster. I highly recommend mentioning the selection method related to the sampling here and citing it with relevant literature.	Thanks for this. We have mentioned the sampling method.	See Lines 267-269
I highly recommend redrawing Figure One for clarity.	Thanks for this. we have redrawn Figure 1	See page 17
•All the references are extremely outdated; I highly recommend using recent resources.	Thanks for this. We disagree with you on this. most of our references are less than 10 years. As you can see, some of the references are theoretical/groundbreaking and thus, the need for its inclusion in the study. We think the necessary references are cited in this study. We have integrated some of the suggested references.	See pages 29-35
• Kindly replace the keywords with these terms and phrases to better suit the article's focus.<br>• For instance, keeping gifted and talented students phrase suits the article however, I would suggest removing (enrichment; acceleration; United Arab Emirates) since the paper does not tackle all the schools in UAE, so we better not generalize the paper's findings.	Thanks for this. We have revised the keywords as follows 'school, gifted and talented students, gifted education, theory of planned behavior, well-being'	See Lines 49-50
• Major language edits need to be performed. Major revisions are needed when it comes to language correctness, kindly either use Grammarly premium or consult	Thanks for this. The paper has been edited professionally.	

external editors to perfect the language to make sure it meets the highest standards.		
• I find this section as being irrelevant and serves an academic purpose<br>" The UAE is an Arab country in Western Asia which is made up of seven emirates: Abu Dhabi, Ajman, Dubai, Fujairah, Sharjah, Ras Al Khaimah, and Umm Al Quwain. Since independence in 1971, the leadership of the country has placed education at the heart of national development [19]. The UAE adopted inclusive education practices at the turn of the 21st century. Specifically, the central government provides educational plans for all students [37]. In 2008, the "School for All" policy was launched by the Ministry of Education, encouraging schools to provide suitable teaching services to high-achieving children [19]. •<br>Instead, the authors could have mentioned a more detailed overview or a comparative approach to how the educational system witnessed massive changes over time	Thanks for this. The section has been deleted.	
• The references do not provide credibility to the topic, as they are extremely outdated and need to be replaced.	Thanks for this. We disagree with you on this. Most of our references are less than 10 years. As you can see, some of the references are theoretical/groundbreaking and thus, the need for its inclusion in the study. We think the necessary references are cited in this study.	
• The research design is not clear.	We have added a study design.	See Lines 252-254
• The methodology section's structure needs to be revised.	We have formatted the method section following your guidelines.	See pages 11 - 18
• " The instrument was made up of three sections. The first section was used to collect demographic". Kindly use academic referencing here; it cannot be stated as a passive statement."	The sentence has been revised as 'The survey was made up of three sections. The first section collected demographic data (gender, age, grade level, and high-ability area(s)), which were included based on a review of the previous literature.'	See Lines 282-284
• Following the standard academic publishing outline, it is	Thanks for this. The paper has been edited professionally.	

advised to follow the following paper structure<br>Introduction<br>Background<br>Literature Review<br>Significance \ Contribution<br>Methods<br>Design<br>Data Collection<br>Data Analysis<br>Discussion<br>Conclusion<br>Recommendations and Future Research<br>It is more advised to clearly identify these sections as mentioned in the above subheading divisions.		
• Major sentence structure revisions are needed.<br>• For instance<br>Google Forms was employed as a virtual platform for data collection because restrictions brought by COVID-19 pandemic. (This sentence is confusing).<br>A better version would be<br>" Google Forms were employed for data collection because of mobility challenges imposed by COVID-19 pandemic."	Thanks for this. The paper has been edited professionally.<br><br>'Google Forms was employed for data collection due to the mobility challenges imposed by the COVID-19 pandemic.'	See Lines 329 – 330
Here, another question arises: when was the study conducted? The pandemic was in 2020-2022, and we are now in the 2024-2025 academic year. As it is stated in the paper<br>The data was collected from May 2022 to July 2022. Where most educational institutions went back to Hybrid and onsite attendance which means there is no clear justification for doing online surveys. You may remove the mention of covid 19 in the above statement and relate it to the reasoning behind selecting such a method.	Thanks for this. In the UAE, schools were closed until the beginning of the 2022/23 academic year. At the time of data collection, there were guidelines for schools to deliver lessons online. The mode of data collection was based on situation that pertains at that time.	
• Major language confusions need to be clarified. For example, consider the examples below	Thanks for this. The paper has been edited professionally.	

" Non-Western countries' contribution of research in gifted education is limited, despite the fact that some have adopted policies to foster the growth of exceptional students."<br>Based on what did you come up with this claim? Which non-western countries?		
• In the methodology section, these need to be clarified<br>150 high school students in one of the seven emirates in the United Arab Emirates to test two hypotheses.<br>Which location? Which school? This cannot be stated as a general statement.	We have revised as follows 'The revised Self-Perception Profile for Children and Opinions About the Gifted and Their Education scales were used to collect data from 150 high school students in Abu Dhabi, one of seven states (emirates) in the United Arab Emirates.'	See Lines 36 – 38
• Criteria bias<br>"The participants of this study were GT high school students nominated by their schools to participate in an enrichment program."<br>This seems a bit unreliable, for instance, what is the selection method used?	Yes. Gifted students are only identified by schools and involved in national programs. This is the viable means to identify or recruit study participants. However, we have noted this in our limitation.	See Lines 524 – 527
• Major language highlights that demand immediate attention<br>• Subject-verb agreement<br>• Kindly note that in many cases, needless capital letters are used, so please revise.	Thanks for this. The paper has been edited professionally.	
**Reviewer 2**		
Th researchers apply ethical consideration. The data are analyzed comprehensively.<br><br>The researchers need to edit the first research question as follows:<br>'What are the differences between..."<br>- Indent the beginning of all paragraphs.	Thanks for this. we formatted the paper according to the journal's guidelines. Research question one has been revised as follows:<br><br>'What are the differences between GT students in demographic variables, attitudes, and self-concepts?'	See page 11
