# Supplementary material for: The nexus between the attitudes and self-concepts of gifted students in an Arab context
Source: PLoS One. 2024 Jun 13;19(6):e0304957. doi: 10.1371/journal.pone.0304957 (PMC11175483; doi:10.1371/journal.pone.0304957)
Supplement: Attachment — Submitted filename: Response to comments.docx [file pone.0304957.s001.docx]

Dear editor,

Thanks for your feedback on our submission. As you can see, the paper has been edited for clarity. More so, we have taken time to incorporate feedback from the reviewers. Below is our response to your feedback on our revised resubmission.

| Comments | Response | Page |
| --- | --- | --- |
| **Reviewer 1** |  |  |
| I highly recommend mentioning the selection method related to the sampling and citing it with relevant literature. The current study drew on Gifted students from private schools as they were running enrichment program for GT students. The decision to include students in private schools was based on the fact that permission ADEK granted permission to include students on their high-ability roster. I highly recommend mentioning the selection method related to the sampling here and citing it with relevant literature. | Thanks for this. We have mentioned the sampling method. | See Lines 267-269 |
| I highly recommend redrawing Figure One for clarity. | Thanks for this. we have redrawn Figure 1 | See page 17 |
| •All the references are extremely outdated; I highly recommend using recent resources. | Thanks for this. We disagree with you on this. most of our references are less than 10 years. As you can see, some of the references are theoretical/groundbreaking and thus, the need for its inclusion in the study. We think the necessary references are cited in this study. We have integrated some of the suggested references. | See pages 29-35 |
| • Kindly replace the keywords with these terms and phrases to better suit the article’s focus.  • For instance, keeping gifted and talented students phrase suits the article however, I would suggest removing ( enrichment; acceleration; United Arab Emirates) since the paper does not tackle all the schools in UAE, so we better not generalize the paper’s findings. | Thanks for this. We have revised the keywords as follows ‘school, gifted and talented students, gifted education, theory of planned behavior, well-being’ | See Lines 49-50 |
| • Major language edits need to be performed. Major revisions are needed when it comes to language correctness, kindly either use Grammarly premium or consult external editors to perfect the language to make sure it meets the highest standards. | Thanks for this. The paper has been edited professionally. |  |
| • I find this section as being irrelevant and serves an academic purpose  “ The UAE is an Arab country in Western Asia which is made up of seven emirates: Abu Dhabi, Ajman, Dubai, Fujairah, Sharjah, Ras Al Khaimah, and Umm Al Quwain. Since independence in 1971, the leadership of the country has placed education at the heart of national development [19]. The UAE adopted inclusive education practices at the turn of the 21st century. Specifically, the central government provides educational plans for all students [37]. In 2008, the “School for All” policy was launched by the Ministry of Education, encouraging schools to provide suitable teaching services to high-achieving children [19]. • Instead, the authors could have mentioned a more detailed overview or a comparative approach to how the educational system witnessed massive changes over time | Thanks for this. The section has been deleted. |  |
| • The references do not provide credibility to the topic, as they are extremely outdated and need to be replaced. | Thanks for this. We disagree with you on this. Most of our references are less than 10 years. As you can see, some of the references are theoretical/groundbreaking and thus, the need for its inclusion in the study. We think the necessary references are cited in this study. |  |
| • The research design is not clear. | We have added a study design. | See Lines 252-254 |
| • The methodology section’s structure needs to be revised. | We have formatted the method section following your guidelines. | See pages 11 - 18 |
| • “ The instrument was made up of three sections. The first section was used to collect demographic”. Kindly use academic referencing here; it cannot be stated as a passive statement.” | The sentence has been revised as ‘The survey was made up of three sections. The first section collected demographic data (gender, age, grade level, and high-ability area(s)), which were included based on a review of the previous literature.’ | See Lines 282-284 |
| • Following the standard academic publishing outline, it is advised to follow the following paper structure  Introduction  Background  Literature Review  Significance \ Contribution  Methods  Design  Data Collection  Data Analysis  Discussion  Conclusion  Recommendations and Future Research  It is more advised to clearly identify these sections as mentioned in the above subheading divisions. | Thanks for this. The paper has been edited professionally. |  |
| • Major sentence structure revisions are needed.  • For instance  Google Forms was employed as a virtual platform for data collection because restrictions brought by COVID-19 pandemic. (This sentence is confusing).  A better version would be  “ Google Forms were employed for data collection because of mobility challenges imposed by COVID-19 pandemic.” | Thanks for this. The paper has been edited professionally.  ‘Google Forms was employed for data collection due to the mobility challenges imposed by the COVID-19 pandemic.’ | See Lines 329 – 330 |
| Here, another question arises: when was the study conducted? The pandemic was in 2020-2022, and we are now in the 2024-2025 academic year. As it is stated in the paper  The data was collected from May 2022 to July 2022. Where most educational institutions went back to Hybrid and onsite attendance which means there is no clear justification for doing online surveys. You may remove the mention of covid 19 in the above statement and relate it to the reasoning behind selecting such a method. | Thanks for this. In the UAE, schools were closed until the beginning of the 2022/23 academic year. At the time of data collection, there were guidelines for schools to deliver lessons online. The mode of data collection was based on situation that pertains at that time. |  |
| • Major language confusions need to be clarified. For example, consider the examples below  “ Non-Western countries’ contribution of research in gifted education is limited, despite the fact that some have adopted policies to foster the growth of exceptional students.” Based on what did you come up with this claim? Which non-western countries? | Thanks for this. The paper has been edited professionally. |  |
| • In the methodology section, these need to be clarified  150 high school students in one of the seven emirates in the United Arab Emirates to test two hypotheses. Which location? Which school? This cannot be stated as a general statement. | We have revised as follows ‘The revised Self-Perception Profile for Children and Opinions About the Gifted and Their Education scales were used to collect data from 150 high school students in Abu Dhabi, one of seven states (emirates) in the United Arab Emirates.’ | See Lines 36 – 38 |
| • Criteria bias  “The participants of this study were GT high school students nominated by their schools to participate in an enrichment program.”  This seems a bit unreliable, for instance, what is the selection method used? | Yes. Gifted students are only identified by schools and involved in national programs. This is the viable means to identify or recruit study participants. However, we have noted this in our limitation. | See Lines 524 – 527 |
| • Major language highlights that demand immediate attention  • Subject-verb agreement  • Kindly note that in many cases, needless capital letters are used, so please revise. | Thanks for this. The paper has been edited professionally. |  |
| **Reviewer 2** |  |  |
| Th researchers apply ethical consideration. The data are analyzed comprehensively.  The researchers need to edit the first research question as follows:  'What are the differences between..."  - Indent the beginning of all paragraphs. | Thanks for this. we formatted the paper according to the journal’s guidelines. Research question one has been revised as follows:  ‘What are the differences between GT students in demographic variables, attitudes, and self-concepts?’ | See page 11 |
