# Supplementary material for: The nexus between the attitudes and self-concepts of gifted students in an Arab context
Source: PLoS One. 2024 Jun 13;19(6):e0304957. doi: 10.1371/journal.pone.0304957 (PMC11175483; doi:10.1371/journal.pone.0304957)
Supplement: Attachment — Submitted filename: Second_response to comments.docx [file pone.0304957.s003.docx]

Dear editor,

Thanks for considering our paper and we appreciate feedback from the reviewers. Below is our point-by-point response to the reviewers’ comments. Thanks once again and looking forward to your feedback.

Comments

Please review your reference list to ensure that it is complete and correct. If you have cited papers that have been retracted, please include the rationale for doing so in the manuscript text, or remove these references and replace them with relevant current references. Any changes to the reference list should be mentioned in the rebuttal letter that accompanies your revised manuscript. If you need to cite a retracted article, indicate the article’s retracted status in the References list and also include a citation and full reference for the retraction notice.

Response

Thanks once again. We have cross-checked and ensured that all references are cited appropriately.

1. If the authors have adequately addressed your comments raised in a previous round of review and you feel that this manuscript is now acceptable for publication, you may indicate that here to bypass the “Comments to the Author” section, enter your conflict of interest statement in the “Confidential to Editor” section, and submit your "Accept" recommendation.

Reviewer #1: All comments have been addressed

Reviewer #2: All comments have been addressed

Response: Thanks for your feedback on our submission.

2. Is the manuscript technically sound, and do the data support the conclusions?

The manuscript must describe a technically sound piece of scientific research with data that supports the conclusions. Experiments must have been conducted rigorously, with appropriate controls, replication, and sample sizes. The conclusions must be drawn appropriately based on the data presented.

Reviewer #1: Yes

Reviewer #2: Yes

Response: Thanks for your feedback on our submission.

3. Has the statistical analysis been performed appropriately and rigorously?

Reviewer #1: Yes

Reviewer #2: Yes

Response: Thanks for your feedback on our submission.

**4. Have the authors made all data underlying the findings in their manuscript fully available?**

The [PLOS Data policy](https://eur03.safelinks.protection.outlook.com/?url=http%3A%2F%2Fwww.plosone.org%2Fstatic%2Fpolicies.action%23sharing&data=05%7C02%7Cmaxwell.p%40uaeu.ac.ae%7Cf24f6bba146f4c2c16a408dc63cd1b1a%7C97a92b044c8743419b08d8051ef8dce2%7C0%7C0%7C638494979896503834%7CUnknown%7CTWFpbGZsb3d8eyJWIjoiMC4wLjAwMDAiLCJQIjoiV2luMzIiLCJBTiI6Ik1haWwiLCJXVCI6Mn0%3D%7C0%7C%7C%7C&sdata=G%2F5C5NFTUC8x%2Fmsb4BFRj2do2ByZguB8iaPxEWl15q4%3D&reserved=0) requires authors to make all data underlying the findings described in their manuscript fully available without restriction, with rare exception (please refer to the Data Availability Statement in the manuscript PDF file). The data should be provided as part of the manuscript or its supporting information, or deposited to a public repository. For example, in addition to summary statistics, the data points behind means, medians and variance measures should be available. If there are restrictions on publicly sharing data—e.g. participant privacy or use of data from a third party—those must be specified.

Reviewer #1: No

Reviewer #2: Yes

Response: We have added a data availability statement (The datasets generated and analysed during the current study are not publicly available due to ethical restrictions but are available from the corresponding author upon reasonable request).

**5. Is the manuscript presented in an intelligible fashion and written in standard English?**

PLOS ONE does not copyedit accepted manuscripts, so the language in submitted articles must be clear, correct, and unambiguous. Any typographical or grammatical errors should be corrected at revision, so please note any specific errors here.

Reviewer #1: No

Reviewer #2: Yes

Response to comments: We have attached an invoice showing the editing of the paper by a reputable language editor. We are confident all errors have been correctly.

**6. Review Comments to the Author**

Please use the space provided to explain your answers to the questions above. You may also include additional comments for the author, including concerns about dual publication, research ethics, or publication ethics. (Please upload your review as an attachment if it exceeds 20,000 characters)

Reviewer #1: I highly recommend you reviewing the language correctness through Grammarly. The paper needs language edits, for this, working on the sentence structure and adjusting the language flaws will enhance the quality of the paper.

Reviewer #2: The authors did the previous edits that were required previously. I do recommend that this paper is ready to be published.

**Response to comments:** We have attached an invoice showing the editing of the paper by a reputable language editor. We are confident all errors have been correctly.
